# Vascular Endothelial Growth Factor (VEGF) Promotes Assembly of the p130Cas Interactome to Drive Endothelial Chemotactic Signaling and Angiogenesis*[Fn FN2]

**DOI:** 10.1074/mcp.M116.064428

**Published:** 2016-12-22

**Authors:** Ian M. Evans, Susan A. Kennedy, Ketevan Paliashvili, Tapesh Santra, Maiko Yamaji, Ruth C. Lovering, Gary Britton, Paul Frankel, Walter Kolch, Ian C. Zachary

**Affiliations:** From the ‡Centre for Cardiovascular Biology and Medicine, Division of Medicine The Rayne Building, University College London, London WC1E 6JJ, United Kingdom;; §Systems Biology Ireland, University College Dublin, Belfield, Dublin 4, Ireland;; ¶Conway Institute of Biomolecular & Biomedical Research, University College Dublin, Belfield, Dublin 4, Ireland;; ‖School of Medicine and Medical Science, University College Dublin, Belfield, Dublin 4, Ireland;; **Centre for Cardiovascular Genetics, Institute of Cardiovascular Science, The Rayne Building, University College London, London WC1E 6JJ, United Kingdom

## Abstract

p130Cas is a polyvalent adapter protein essential for cardiovascular development, and with a key role in cell movement. In order to identify the pathways by which p130Cas exerts its biological functions in endothelial cells we mapped the p130Cas interactome and its dynamic changes in response to VEGF using high-resolution mass spectrometry and reconstruction of protein interaction (PPI) networks with the aid of multiple PPI databases. VEGF enriched the p130Cas interactome in proteins involved in actin cytoskeletal dynamics and cell movement, including actin-binding proteins, small GTPases and regulators or binders of GTPases. Detailed studies showed that p130Cas association of the GTPase-binding scaffold protein, IQGAP1, plays a key role in VEGF chemotactic signaling, endothelial polarization, VEGF-induced cell migration, and endothelial tube formation. These findings indicate a cardinal role for assembly of the p130Cas interactome in mediating the cell migratory response to VEGF in angiogenesis, and provide a basis for further studies of p130Cas in cell movement.

Vascular Endothelial Growth Factor (VEGF^1^ or VEGF-A) is essential for angiogenesis during development and in the pathogenesis of human pathologies including cancer and eye diseases (1, 2). VEGF stimulates its diverse cellular functions in endothelial cells through high affinity binding to two tyrosine kinase receptors, VEGF receptor 1 (VEGFR1 or Flt-1) and VEGFR2 (or KDR), though VEGFR2 is largely responsible for functional VEGF-triggered signal transduction (3, 4). VEGFR2 is activated through ligand-stimulated receptor dimerization and trans(auto)phosphorylation of multiple tyrosine residues in the cytoplasmic domain (5, 6), inducing multiple signaling events followed by early and long-term cellular effects including production of the vasoactive mediators, prostacyclin and nitric oxide, increased cell survival, migration, proliferation and angiogenesis (4, 6–14). Neuropilin-1 (NRP1) is a coreceptor for VEGF in endothelial cells, and is essential for embryonic angiogenesis and vascular development (15–17). NRP1 is thought to act as a coreceptor for VEGF by forming complexes with VEGFR2, which enhance intracellular signaling, cell migration, and angiogenesis (18). *In vivo*, VEGF binding to NRP1 appears to be largely dispensable for embryonic vascular development, but is required for perinatal retinal vascularization, and for post-natal angiogenesis in pathophysiological settings (19).

We previously identified a key role for the p130Cas adaptor protein in mediating VEGFR2/NRP1-dependent endothelial cell migration stimulated by VEGF and PDGF-induced migration in vascular smooth muscle cells (20, 21). A critical role for p130Cas, encoded by the *Bcar1* gene, in cardiovascular development is supported by the phenotype of *Bcar1* null mice (22), which die *in utero* with severe defects in the heart and vasculature seen at embryonic days (E) 11.5–12.5 when p130Cas is predominantly expressed in the cardiovascular system of wild type mice. In human endothelial cells, VEGF rapidly stimulates p130Cas tyrosine phosphorylation in a NRP1-dependent manner, and VEGF-induced endothelial cell migration and angiogenesis are inhibited by either p130Cas-targeted siRNA or by overexpression of a p130Cas mutant that is nonphosphorylatable at tyrosine residues in the substrate domain (20).

p130Cas is a crucial node in chemotactic signaling in diverse cells types, able to interact with multiple binding partners implicated in the regulation of cell migration, including Crk (C10 regulator of kinase), p60 c-Src, FAK and protein tyrosine kinase 2 (PYK2) (23, 24). p130Cas binding to intracellular interactors mediates activation of downstream effectors such as the guanine-exchange factors (GEFs) DOCK180-ELMO (Engulfment and cell motility) and C3G, which in turn enhance the activity of the small GTPases Rac and Rap (25–28), essential for actin reorganization in lamellipodia and membrane ruffle formation. Hitherto, p130Cas protein-protein interactions have been identified on a case by case basis from candidate-based studies using coimmunoprecipitation experiments (23, 24). The structural features of p130Cas that fit it to the role of a polyvalent hub for protein-protein interactions, and its essential functions in cell motility, in cardiovascular development and in endothelial VEGF signaling, revealed by mouse genetic and cellular studies, make p130Cas a particularly attractive candidate for interactome analysis using an unbiased proteomic and systems biology approach. We therefore sought to define the p130Cas-interacting partners in endothelial cells relevant for VEGF-driven chemotaxis using mass spectrometry combined with bioinformatic analysis of p130Cas-associated proteins. Our findings reveal that VEGF stimulation enriched the p130Cas interactome in several major classes of protein involved in cell movement or cellular processes linked to cell motility, including many novel p130Cas-interacting proteins. Targeted studies on selected components of the p130Cas interactome supported the initial proteomics analysis and identified novel mediators of endothelial cell motility and angiogenesis. This is the first proteomic analysis of the p130Cas interactome, and its results demonstrate a key role for p130Cas and its interactome in VEGF angiogenic signaling in endothelial cells.

## MATERIALS AND METHODS

### 

#### 

##### Immunoprecipitation

HUVECs cultured on 10 cm dishes, which had been infected with adenoviruses (Ad) encoding either wild-type (WT) p130Cas (Ad.p130Cas), or a p130Cas mutant with 15 tyrosine residues mutated to phenylalanine (Ad.p130Cas15F), were treated with VEGF (0,10, 30 and 60 min) in three independent experiments, and then lysed with 10 mm Tris-HCl pH 7.4, 50 mm NaCl, 5 mm EDTA, 1% Triton-X100, Complete^TM^ protease inhibitor mixture EDTA free and phosphatase inhibitors (Sigma, Dorset, UK). Lysates were centrifuged at 20,000 × *g* for 10 min to remove insoluble debris, precleared by mixing with A/G agarose beads in lysis buffer for 1 h with constant mixing, and were then used for immunoprecipitation (lysate from one 10 cm dish of cells per immunoprecipitation) with p130Cas antibody (1:50 dilution) for 16 h. In addition, HUVECs which had been infected with Ad.p130Cas (WT), were treated with VEGF (0,10, 30, and 60 min) in three independent experiments, lysed as above, and used for immunoprecipitation with isotype-matched control antibody (mouse IgG; 1:10 dilution; Santa Cruz Biotechnology, Dallas, TX, catalog number sc-2025) for 16 h. All immunocomplexes were captured with Protein A/G PLUS agarose beads (Santa Cruz) for 1 h. Immunoprecipitates were washed three times with lysis buffer, and separated on SDS-PAGE gels. Immunoprecipitates that were used for mass spectrometry were washed three times with lysis buffer and twice with PBS to remove detergent. All above procedures were carried out on ice or at 4 °C. Beads were stored on dry ice, and immunoprecipitated proteins were eluted in a 2-step process (29). First, beads were incubated with 60 μl eluting buffer 1 (2 m urea, 50 mm Tris-HCl pH7.5, and 5 μg/ml trypsin (modified sequencing grade trypsin); Promega, Southampton, UK) at 27**°**C for 30 min under gentle agitation. Then, samples were centrifuged and the supernatants were placed in a fresh tube. For the second elution step 2 × 25 μl elution buffer 2 (2 m urea, 50 mm Tris-HCl pH7.5, and 1 mm DTT) was added to the beads. All supernatants were combined and allowed to digest overnight at room temperature. The following morning, samples were alkylated with 20 μl iodoacetamide (5 mg/ml) for 30 min in the dark. This reaction was stopped with 1 μl of 100% trifluoroacetic acid (TFA), and the sample (∼110 μl) was immediately loaded onto equilibrated C18 StageTips, prepared as described previously (30). Samples were added to the activated tips, desalted using 2 × 50 μl of 1% TFA solution, and eluted with 2 × 25 μl of 50% ACN and 0.1% TFA solution. Final eluates were combined and concentrated using a CentriVap concentrator (Labconco, Fort Scott, KS), resuspended in 12 μl 0.1% TFA and analyzed by mass spectrometry.

##### Mass Spectrometry and Data Analysis

The samples prepared from immunopreciptates which had been obtained from three independent cultures of HUVECs, each treated with VEGF for different times, were analyzed on a Q-Exactive mass spectrometer (Thermo Fisher Scientific, Loughborough, UK) connected to a Dionex Ultimate 3000 (RSLCnano) chromatography system (Thermo Fisher Scientific) incorporating an autosampler. A total of 5 μl of tryptic peptides from each sample was loaded onto a homemade column (100 mm length, 75 μm inside diameter), packed with 1.9 μm ReprosilAQ C_18_ (Dr. Maisch GMBH, Ammerbuch-Entringen, Germany) and separated by an increasing acetonitrile gradient using a 40 min reverse-phase gradient (from 3–32% Acetonitrile) at a flow rate of 250 nL/min. The mass spectrometer was operated in positive ion mode with a capillary temperature of 220**°**C and a potential of 2000 V applied to the capillary. All data were acquired with the mass spectrometer operating in automatic data-dependent switching mode. A high-resolution MS scan (350–2000 Da) was performed using the Orbitrap to select the 12 most intense ions for fragmentation and MS/MS analysis. The MaxQuant software suite (v 1.5.0.25) containing the in-built Andromeda search engine was used to identify the proteins from a human database (Uniprot HUMAN, release 2012_01) containing 20,242 entries, and determine their relative concentration by label-free quantification. For database searches, the precursor mass tolerance was set to 20 ppm for first searches and 4.5 ppm for main Andromeda search. MaxQuant default parameters were used with the exception of minimum ratio count and LFQ minimum ratio count set to 1. The search included a fixed modification of Carbamidomethyl (C) and variable modifications of Oxidation (M);Acetyl (Protein N-term). A fully tryptic specific search was used. The maximum number of missed cleavages was set at 2 and minimum peptide length was set to 7 amino acids. An FDR of 0.01 was set for peptide and protein identifications. Match between runs was selected with a matching time window of 0.7 min and alignment time window of 20 min. The presence of reverse and contaminant identifications was removed from the dataset. For quantification purposes LFQ intensity values from MaxQuant were used for statistical analysis. All samples were analyzed in technical duplicates (labeled “a” and “b” in supplemental tables and biological triplicates, and as such every MS file was considered separately for the subsequent statistical analysis. Full details of peptide sequences, and protein identifications (including accession numbers) can be found in supplemental Tables S1 and S2. The mass spectrometry proteomics data have been deposited to the ProteomeXchange Consortium via the PRIDE (31) partner repository with the dataset identifier PXD003902 (http://www.ebi.ac.uk/pride/archive/).

##### Determining VEGF Induced Interactomes of Wild-type and Mutant p130cas

Log-transformed protein intensities of p130Cas immunoprecipitates from HUVECs transduced with Ad expressing either p130Cas (Ad.p130Cas), or p130Cas15F (Ad.p130Cas15F, mutant) and which had been VEGF treated (0, 10, 30 and 60 min) were compared with the corresponding isogenic controls samples from HUVECs transduced with Ad.p130Cas (WT), and which had been VEGF treated (0, 10, 30 and 60 min). The comparison was performed using BDiffProt algorithm (32), which identified differentially expressed proteins between the control (isogenic control, transduced with Ad.p130Cas) and noncontrol (p130cas immunoprecipitates, transduced with Ad.p130Cas or Ad.p130Cas15F) samples. We chose BDiffProt because it was shown to have superior performance over other methods that are commonly used to find differentially expressed proteins. Additionally, it does not require missing data points to be inputed and performs well on both normal and nonnormal protein intensity data (32). However, because BDiffProt has not previously been used to analyze data from affinity purification combined with Mass-Spectrometry (AP-MS) we confirmed the suitability of this algorithm by using it to analyze a previously published AP-MS derived data set (33). Two sets of samples, containing empty vectors (negative controls) and PHD3 baits were analyzed. BDiffProt, combined with a fold change threshold of 2, identified 1473 proteins to have differential intensities between these two types of samples. In the original publication (33), 1279 proteins were detected to be differentially expressed using *t* test and the same fold-change threshold. Approximately 91% of the proteins identified in the original publication were also identified by the BDiffProt analysis (supplemental Fig. S1). The details of the BDiffProt output and Bayesian analysis (including the proteins excluded from further analysis) are given in supplemental Tables S3 and S4 respectively. Proteins which were identified to be differentially expressed by BDiffProt algorithm and had at least 2-fold increase between wild-type/mutant p130cas transduced samples and the isogenic controls at each time of VEGF treatment were considered to be potential p130cas interactors. Proteins that were not selected in the above process were discarded from further analysis. For the potential p130cas interactors, the intensities in samples treated with VEGF for 10, 30 and 60 min were further compared with the intensities in untreated samples (0 min VEGF treatment) using BDiffProt algorithm. Proteins that were found to be differentially expressed and had at-least 2-fold increase in intensities between untreated and VEGF treated samples were considered to form the VEGF induced interactome of wild-type and mutant p130cas. Fold-changes were calculated by first replacing the missing intensities with the minimum intensity in the dataset (34, 35), calculating the average intensities in each of 10, 30, and 60 min VEGF treated and untreated group of samples, and then dividing the average intensity of each treated group by that of the untreated group.

##### Gene Ontology(GO) Enrichment Analysis

The gene ontology enrichment of each cluster of interactors were analyzed using the Gene Ontology Consortium's enrichment analysis tool (http://geneontology.org/page/go-enrichment-analysis).

##### Cell Polarity Assay

HUVECs were plated onto coverslips to form a confluent monolayer. The center of the coverslip was scoured with a pipette tip, removing cells and leaving a gap. Cells were incubated for 90 min to allow them to polarize toward the scratch, then fixed in 4% PFA. Cells were stained with an anti-GM130 (Golgi marker) antibody. Actin and nuclei were visualized using TRITC-phalloidin and DAPI, respectively.

##### Other Methods

Cell culture, adenoviral construction and infection, recombinant protein generation and pulldowns, immunofluorescent staining and confocal imaging, focal adhesion isolation, and assays of chemotaxis and angiogenesis in cocultures, were performed as previously described (18, 20, 36) and are detailed in supplementary materials.

##### Experimental Design and Statistical Rationale

For quantification purposes LFQ intensity values from MaxQuant were used for statistical analysis. All samples were analyzed in technical duplicates (labeled “a” and “b” in supplemental tables and biological triplicates (labeled 1–3 in supplemental Tables S1 and S2), and as such every MS file was considered separately for the subsequent statistical analysis.

The data displayed in the graphs are means, with error bars representing the standard error of the mean (s.e.m.), n values for each experiment are detailed in the appropriate figure legends. Statistical analysis was performed by two-way analysis of variance with a Bonferroni post-test. A *p* value of <0.05 was considered significant.

## RESULTS

### 

#### 

##### Identification of the Endothelial Cell p130Cas Interactome and Dynamic Regulation by VEGF

**Fig. 1.**
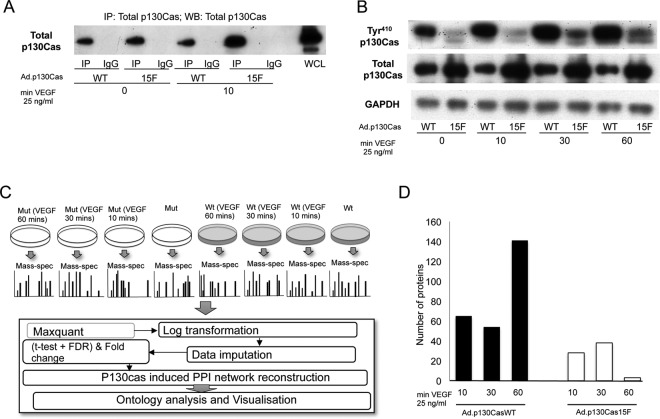
**Biochemical and computational workflow for determining the interactomes of mutated and wild type p130cas, before and after VEGF stimulation.** HUVECs were infected with Ad.p130CasWT or Ad.p130Cas15F for 48h. Cells were then incubated for 18 h in EBM with 0.5% FBS followed by stimulation with or without VEGF for the times indicated. Cells were then lysed and p130Cas was immunoprecipitated. *A*, Representative blot demonstrating immunoprecipitation of a band corresponding to the p130Cas detected in whole cell lysates (*WCL*), but not with the IgG control. *B*, Representative blot of WCLs showing that VEGF treatment increases the amount of tyrosine phosphorylated (Tyr^410^) p130Cas in Ad.p130CasWT infected cells but not in cells overexpressing p130Cas15F. *C*, Overview of the experimental procedure and downstream analysis. Briefly, confluent HUVECs were infected with either Ad.p130CasWT or Ad.p130Cas15F for 48h, incubated overnight in EBM/0.5% FBS followed by stimulation with VEGF for 0, 10, 30, or 60 min. Cells were lysed and p130Cas immunoprecipitated in three independent experiments. Immunoprecipitated proteins were prepared and identified by LC-MS/MS prior to reconstruction of the p130Cas interactome. See Materials and Methods & supplemental material for further details. *D*, Number of proteins showing increased association with p130Cas after VEGF stimulation for the times indicated of cells overexpressing Ad.p130CasWT (*black bars*) or Ad.p130Cas15F (*open bars*). Numbers shown indicate proteins identified in all 3 experiments at each treatment time.

To identify the VEGF-responsive p130Cas interactome in endothelial cells mass spectrometry (MS) analysis was performed on p130Cas immunoprecipitates (IPs) prepared from cells treated with VEGF for 10, 30, and 60 min in three independent experiments (Fig. 1*A*). In order to enrich the signal for p130Cas-interacting proteins to a detectable level for label-free MS, we over-expressed wild-type (WT) p130Cas in endothelial cells, and to determine which interactions were specific for tyrosine phosphorylated p130Cas, we additionally compared immunoprecipitates prepared from cells over-expressing p130CasWT with those expressing a nonphosphorylatable p130Cas mutant (p130Cas15F), in which all 15 potential tyrosine phosphorylation sites in the substrate domain were mutated to phenylalanine (20). We also compared immunoprecipitates prepared from cells overexpressing p130CasWT or p130Cas15F with immunoprecipitates prepared from cells overexpressing p130CasWT using an isotype-matched control antibody. VEGF stimulated a striking increase in p130Cas tyrosine phosphorylation in HUVECs expressing Ad.p130CasWT, which was suppressed in HUVECs expressing Ad.p130Cas15F (Fig. 1*B*). Similar amounts of total p130Cas were immunoprecipitated from cells expressing p130CasWT and p130Cas15F (Fig. 1*A*) as confirmed by mass spectrometry (supplemental Fig. S2).

**Fig. 2.**
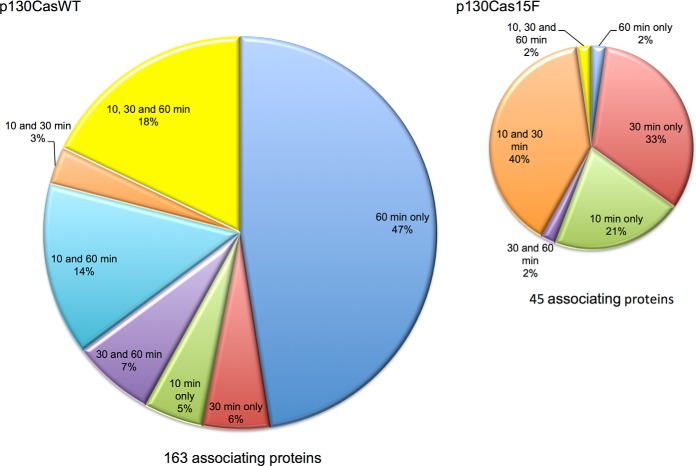
**Comparison of the dynamic interactomes of wildtype and mutant p130cas during VEGF stimulation.** Pie charts showing the percentage of p130Cas-interacting proteins at each VEGF treatment time point and between times for the WT and mutant p130Cas overexpressing HUVECs. The diameter of WT and mutant pies is proportional to the interactome size.

MS analysis identified proteins in the p130Cas interactome that were enriched in IPs prepared from cells in all three independent experiments, both expressing p130CasWT as compared with cells expressing p130Cas15F, and in IPs prepared from cells expressing p130CasWT that had been treated with VEGF for 10, 30 or 60 min as compared with untreated cells (Fig. 1*C*). VEGF induced significant changes in the intensities of 163 and 45 proteins, respectively, in WT and mutant p130cas-expressing cells (Figs. 1*D* and 2, supplemental Tables S5 and S6). These proteins constitute the VEGF induced interactomes of p130CasWT and p130cas15F, respectively. The largest number of VEGF induced p130casWT interactors were identified after 60 min VEGF treatment, and the second largest group were persistent interactors, that is their intensities increased after 10 min VEGF treatment and did not change significantly between 10, 30, and 60 min treatment (Fig. 2). Components of the p130casWT interactome showed different temporal patterns of interaction: persistent interactors, delayed interactors of p130casWT, which had higher intensities at 30 and 60 min after VEGF stimulation than at 10 min; late interactors, with higher intensities at 60 min after VEGF treatment than at 10 or 30 min; returning interactors which had higher intensities at 10 and 60 min following VEGF treatment than at 30 min, and early interactors of p130casWT, *i.e.* have higher intensities at 10 min following VEGF treatments than 30 or 60 min.

The p130cas15F interactome was much smaller, the largest group in this interactome comprising transient interactors, exhibiting higher intensities at 10 and 30 min of VEGF treatment than at 60 min with few proteins early or persistent interactors (Fig. 2). Few proteins were found in both the p130casWT and p130cas15F interactomes at any of the times of VEGF treatment, indicating little overlap in the WT and mutant p130Cas interactomes.

##### Gene Ontology Analysis of the p130Cas Interactome

These findings indicated that VEGF causes a marked, time-dependent increase in complexation of p130Cas with a large number of cellular proteins, and that formation of the VEGF-dependent p130Cas interactome is strongly dependent on tyrosine phosphorylation of the p130Cas substrate domain. In order to visualize specific interactome changes under different conditions, we reconstructed a generic p130Cas interaction network from the Gene Ontology Consortium database and our MS results, and functionally interpreted the p130Cas interactomes by quantifying the number of proteins associated with specific gene ontology (GO) annotations that significantly changed depending on the conditions.

**Fig. 3.**
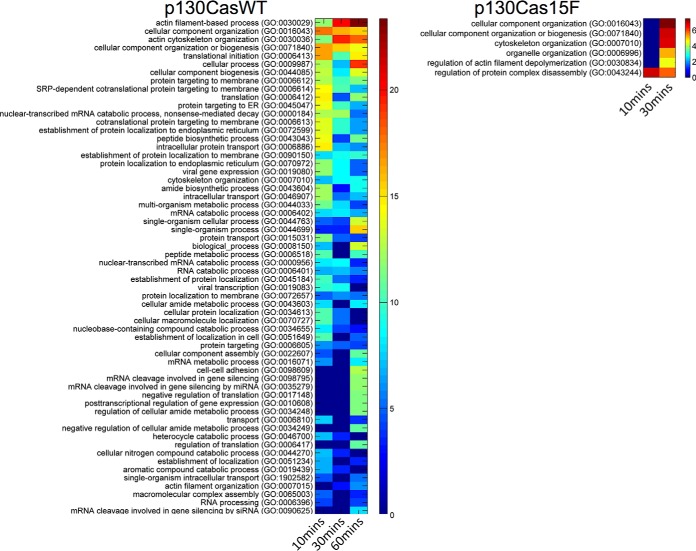
**Comparison of enriched GO terms in the VEGF induced WT and mutant p130Cas interactomes.** Enriched GO terms for the WT (*left hand panel*) and mutant (*right hand panel*) p130Cas interactomes were determined using the Gene Ontology Consortium's enrichment analysis tool. There were no enriched GO terms in the mutant p130cas interactors at 60 min, therefore, only those enriched GO terms in mutant p130cas at 10 and 30 min are shown. The color scale indicates fewer proteins associated with GO term (*blue*) and increasing proteins associated with GO term denoted by red shading.

Clustering analysis revealed dynamic changes in the VEGF induced p130casWT and interactome following VEGF treatment (Fig. 3). GO enrichment analysis revealed that the functional groups exhibiting the highest intensities in the p130casWT interactome at 10, 30 and 60 min following VEGF treatment included proteins involved in actin filament-based processes and actin cytoskeletal organization. Other GO groups featuring in the p130CasWT interactome were those associated with cellular component organization and biogenesis, protein translation, intracellular vesicular protein transport, and regulation of mRNA processing and expression (Fig. 3). Among proteins displaying a persistent and/or delayed interaction with p130casWT, were cytoskeletal proteins, actin-binding proteins and regulators of actin filament assembly, proteins with established roles in cell migration, and several proteins implicated in angiogenesis (supplemental Table S5). These included several proteins with known roles in the regulation of cell migration, and/or endothelial cell function, VEGF signaling, or angiogenesis, including IQGAP1, Profilin-1, CDC42 binding protein kinase beta (CDC42BPB or myotonic dystrophy-related CDC42-binding kinase (MRCKβ)), formin homology domain proteins (FMNL or FHOD 1 and 3), and RhoGEF and PH domain containing 5 (FGD5). In addition, VEGF also increased p130casWT interaction with other proteins with established roles in actin filament organization or in cell migration in other cell types, including aldolase-A, microtubule associated monooxygenase, calponin and LIM domain containing 2 (MICAL2), spire type actin nucleation factor 1 (SPIRE1), Slit-Robo Rho GTPase activating protein 1 (SRGAP1), cysteine and glycine rich proteins 1 and 2 (CSRP1 and 2), tyrosine 3-monooxygenase/tryptophan 5-monooxygenase activation protein epsilon (YWHAE or 14–3-3 epsilon). VEGF also enriched the p130casWT interactome in a significant number of proteins with roles in regulation of post-transcriptional mRNA processing, or in vesicular protein trafficking. In contrast, the mutant p130cas15F interactome contained far fewer enriched GO terms than the p130casWT, and no GO enrichment was detected at 60 min VEGF treatment (Fig. 3). Together, these results support a central role for p130Cas and its interactome in VEGF regulation of actin cytoskeletal organization and motility, and identifies novel p130Cas-interacting proteins with potential roles in VEGF and p130Cas mediated cellular functions.

##### Functional Role of p130Cas Interactome Constituents in Endothelial Cells

**Fig. 4.**
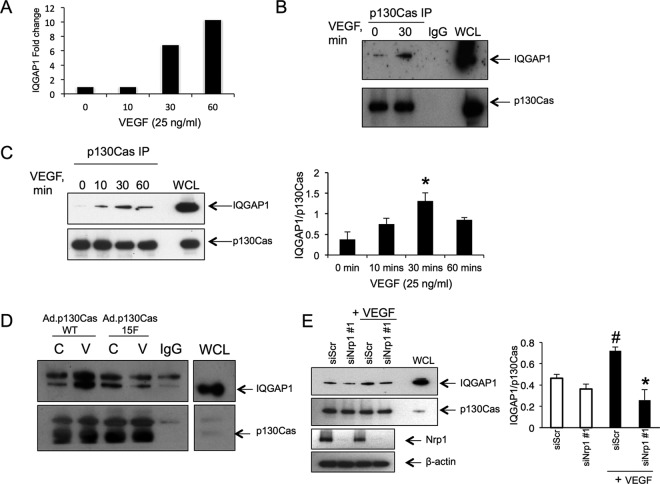
**IQGAP1 associates with p130Cas in a VEGF-dependent manner and also requires p130Cas tyrosine phosphorylation.** *A*, Fold change in IQGAP1 association with VEGF treatment in HUVECS infected with Ad.p130CasWT as determined by the LFQ intensities. *B*, *C*, Cells were incubated in EBM/0.5% serum for 18 h prior to treatment with VEGF for the times indicated. Cells were lysed and immunoprecipitated with anti-p130Cas antibody and immunoblotted with antibodies to p130Cas and IQGAP1. Coimmunopreciptated IQGAP1 in *C* from 3 independent experiments was quantified by densitometry and normalized to total immunopreciptated p130Cas using ImageJ; *p < 0.05 *versus* 0 min time point, *n* = 3. *D*, HUVECs were infected with either Ad.p130CasWT or Ad.p130Cas15F for 48 h. Adenovirus infected cells, in parallel with uninfected cells, incubated in EBM/0.5% serum for 18 h followed by stimulation with or without 25 ng/ml VEGF for 30 min. Cells were lysed and immunoprecipitated with anti-p130Cas antibody and immunoblotted with antibodies to p130Cas and IQGAP1. Blots shown are representative of at least 2 independent experiments. *E*, Cells were transfected with 200 nm siNRP1 or 200 nm siScr for 48h, incubated in EBM/0.5% serum for a further 18 h, then stimulated with or without VEGF for 30 min, lysed and immunoprecipitated with anti-p130Cas antibody and immunoblotted with antibodies to p130Cas and IQGAP1. Quantification of coimmunoprecipitated IQGAP1 is shown below. ^#^*p* < 0.05 *versus* siScr minus VEGF, *p < 0.01 *versus* siScr plus VEGF, *n* = 3.

In order to infer with greater confidence a functional role for individual protein associations with p130Cas in VEGF signaling relevant for cell movement, we next examined associations, and their regulation by VEGF treatment, of selected interactome constituents with endogenous p130Cas expressed at a physiological level. We confirmed endogenous p130Cas association with three interactors with known roles in cell migration identified in our interactome analysis, IQGAP1, Profilin-1, and MRCKβ by p130Cas immunoprecipitation followed by Western blot with antibodies specific to these interactors (supplemental Fig. S3, Fig. 4).

The scaffold protein, IQGAP1, was strongly increased in the WTp130Cas interactome by VEGF treatment (Fig. 4*A*). IQGAP1 is implicated in the regulation of cell motility through interactions with cell adhesion molecules, such as E-cadherin, and with the small GTPases, Rac1 and Cdc42, which are involved in lamellipodia protrusion (36, 37), and is reported to mediate VEGF-dependent generation of reactive oxygen species and endothelial migration via Akt signaling, and angiogenesis (38–40). However, the interaction between p130Cas and IQGAP1 has not previously been identified, and it is unclear how the scaffold function of IQGAP1 mediates its functional roles in endothelial cells. Western blot of immunoprecipitates of endogenous p130Cas from endothelial cells with antibody specific to IQGAP1, confirmed the VEGF-regulated association with p130Cas originally found in p130Cas-overexpressing cells (Fig. 4*B*). We therefore examined the interaction of IQGAP1 and p130Cas in VEGF signaling and endothelial migratory cell functions in greater detail as an exemplar of the functional importance of the p130Cas interactome.

Immunoprecipitation of endogenous p130Cas followed by IQGAP1 Western blot showed that although some constitutive coimmunoprecipitation of these proteins occurred, VEGF significantly increased association of p130Cas with IQGAP1 (Fig. 4*B*, 4*C*). VEGF stimulated the association between endogenous IQGAP1 and p130Cas proteins in a time-dependent manner with a detectable increase at 10 min, and significant enhancement at 30 min (Fig. 4*C*). In HUVECs overexpressing p130CasWT, VEGF increased the p130Cas association with IQGAP1, but this stimulation was completely blocked by overexpression of the nontyrosine phosphorylatable p130Cas15F mutant (Fig. 4*D*), indicating that interaction between these proteins was dependent on p130Cas tyrosine phosphorylation, consistent with our proteomics analysis. The importance of p130Cas tyrosine phosphorylation for the relay of VEGF signaling to IQGAP1 was further tested by examining the effect of p130Cas depletion on VEGF-stimulated IQGAP1 association with tyrosine phosphorylation (41). IQGAP1 immunoprecipitation with anti-phosphotyrosine antibody in unstimulated endothelial cells was low, but was markedly increased by VEGF treatment for 15 min, an effect that was abolished by p130Cas knockdown (supplemental Fig. S4*A*). Furthermore, inhibition of Src, which is an important mediator of p130Cas tyrosine phosphorylation, using the selective inhibitor PP2, strongly reduced VEGF-stimulated IQGAP1 anti-phosphotyrosine immunopreciptation (supplemental Fig. S4*B*). In contrast, inhibition of Focal adhesion kinase (FAK) and the related kinase PYK2, using PF-573,228, had no effect on VEGF-induced IQGAP1 association with tyrosine phosphorylation (supplemental Fig. S4*B*).

The interaction between p130Cas and IQGAP1 was further investigated by examining interactions between recombinant full-length IQGAP1 protein fused to GST (supplemental Fig. S5*A*), and endogenous p130Cas. Incubation of GST-IQGAP1 with HUVEC lysates resulted in p130Cas pull-down, and this interaction was not affected by VEGF treatment of the cells, indicating that endogenously expressed p130Cas associates with IQGAP1 (supplemental Fig. S5*B*). The interaction of p130Cas with IQGAP1 was further investigated in pull-down experiments with recombinant p130Cas GST-fusion proteins containing either the carboxy-terminal or amino-terminal portions of p130Cas. Immunoblot of IQGAP1 in endothelial lysates following incubation with p130Cas GST-fusion proteins showed that IQGAP1 was selectively pulled down only by the p130Cas amino-terminal region comprising the tyrosine phosphorylated kinase substrate domain, the SH3 domain and the proline-rich region (supplemental Fig. S5*C*).

NRP1 is important for mediating VEGF signaling via increased p130Cas tyrosine phosphorylation and is strongly implicated in VEGF regulation of directed endothelial cell movement (18–20). The role of NRP1 in relaying VEGF-induced signals to larger multimolecular complexes involved in actin re-organization and cell movement is presently unclear. Depletion of NRP1 using two specific NRP1-targeted siRNAs inhibited the VEGF-induced p130Cas association with IQGAP1 (Fig. 4*E* and supplemental Fig. S6), indicating that VEGF stimulates the interaction between IQGAP1 and p130Cas via an NRP1-dependent pathway.

##### Role of the p130Cas/IQGAP1 Association in VEGF-induced Focal Adhesion Assembly, Migration, and Angiogenesis

VEGF stimulates a redistribution of p130Cas in endothelial cells to focal adhesions (FA) and the membrane where it colocalizes with F-actin (supplemental Fig. S7*A*) (20). VEGF also caused a marked change in the cellular distribution of IQGAP1 from a preponderantly diffuse granular cytoplasmic staining to a pattern of discrete staining at the plasma membrane and increased costaining of IQGAP1 with vinculin in FAs and with F-actin, particularly in membrane ruffles (supplemental Fig. S7*A*, S7*B*).

**Fig. 5.**
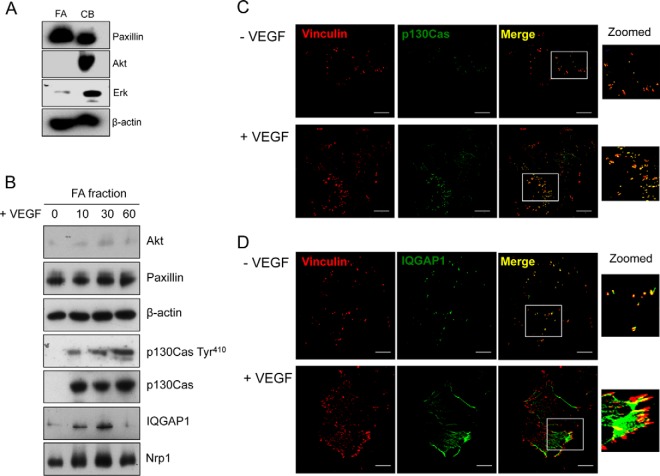
**VEGF induces localization of p130Cas and IQGAP1 into focal adhesions.** Focal adhesion (*FA*) isolates were prepared as detailed in Materials and Methods and validated by Western blot and immunofluorescent analysis. *A*, Representative blots of lysates from either the cell body (*CB*) or FA fractions immunoblotted with the indicated antibodies. Paxillin is expressed at higher levels in focal adhesions whereas Akt and Erk expression is largely limited to the cell body fraction. β-actin was used to confirm equal loading. *B*, Representative blots of isolated FAs after VEGF treatment. HUVECs were plated onto fibronectin and made quiescent by overnight incubation in EBM/0.5% FBS. Cells were stimulated for 0 to 60 mins with 25 ng/ml VEGF before FA isolation. *C*, *D*, FAs were prepared from HUVECs adhering to fibronectin-coated coverslips, and stained with antibodies to vinculin (red) and either p130Cas or IQGAP1 (*C*, *D* respectively; *green*). Images shown are representative of at least 3 different experiments.

Because assembly of multimolecular complexes at FAs plays a key role in regulating cell adhesion to extracellular matrix and cell movement, we examined the VEGF-dependent recruitment of IQGAP1 to FAs in isolated FAs prepared from fibronectin adherent cells using hydrodynamic force (36). Western blotting and immunostaining showed that isolated FAs were enriched in the major FA-associated protein, paxillin, whereas Akt was only weakly detected in FAs and was enriched in the cell body fraction (Fig. 5*A*, supplemental Fig. S8). FAs in control cells contained some p130Cas, but VEGF markedly and rapidly increased p130Cas FA localization, as judged by Western blot of FA lysates (Fig. 5*B*). Increased p130Cas expression in FAs was accompanied by increased phosphorylation of p130Cas at Y410. Concomitant with expression of total and phosphorylated p130Cas in FAs, VEGF also markedly increased colocalization of p130Cas and vinculin in isolated FAs as determined by immunofluorescent staining (Fig. 5*C*). VEGF promoted IQGAP1 recruitment to FAs with a similar time-course. IQGAP1 exhibited some localization to FAs in control cells, but VEGF caused a striking redistribution of IQGAP1 to membrane ruffles at the cell periphery, that also colocalized with FAs (Fig. 5*D*).

**Fig. 6.**
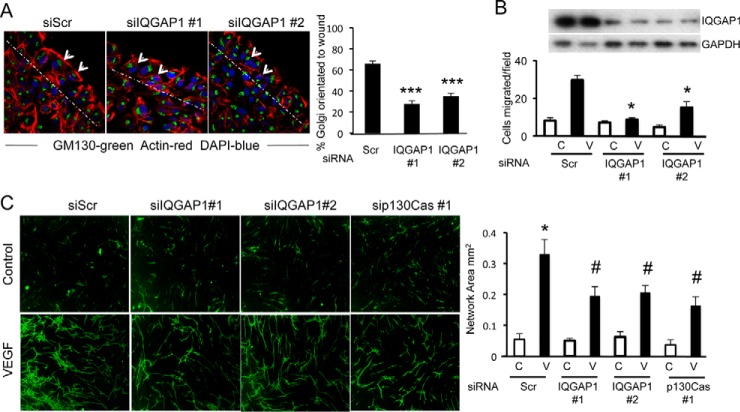
**IQGAP1 is required for VEGF-induced cell polarization, migration and angiogenesis.** *A*, HUVECS were transfected with two different siRNAs targeting IQGAP1 at a concentration of 200 nm, or 200 nm of siScr, for 72h and plated onto coverslips to form a confluent monolayer. The center of the coverslip was scored with a pipette tip, removing cells and leaving a gap. Cells were incubated for 90 min to allow them to polarize toward the scratch, then fixed in 4% PFA. Cells were stained with antibodies to Actin (red) and GM130 (Golgi marker; *green*) and nuclei were visualized using DAPI (blue). Cells were determined to be polarized when the golgi were orientated to face the scratch. *Arrowheads* indicate examples of nonpolarized cells. Polarized and nonpolarized cells were counted up to 40 μm from the scratch (indicated by the *white dotted line*) and expressed as a percentage of cells orientated toward the scratch. One-way ANOVA indicates a significant reduction in the percentage of cells polarized toward the scratch in the IQGAP1 knockdown samples (***p < 0.0005 for both IQGAP1 siRNAs). *B*, HUVECS were transfected with two different siRNAs targeting IQGAP1 at a concentration of 200 nm, or 200 nm of siScr for 72h and cell migration determined in response to 25 ng/ml VEGF. Values (n ≥ 3) are means ± s.e.m, expressed as the number of cells migrating per field; *p < 0.05 compared with siScr. Inset panel shows representative blots of siRNA mediated knockdown of IQGAP1. *C*, HUVECS were transfected with two different siRNAs targeting IQGAP1 at a concentration of 200 nm, or 200 nm of siScr for 48 h. Cells were trypsinized and 1 × 10^4^ cells were applied to a confluent monolayer of human dermal fibroblasts in EBM supplemented with 1% FBS and with or without 25 ng/ml VEGF. Cocultures were incubated for 7 days, with a change of media after 4 days, before being fixed in ethanol. Endothelial cells were stained with anti-VWF antibody followed by an Alexafluor 488 conjugated secondary antibody. Tube formation was visualized by scanning in an Incucyte Zoom and parameters of tube formation were determined using the Incucyte Zoom Angiogenesis software package. Representative images of endothelial tube formation after 7 days coculture with human dermal fibroblasts either in the absence (*upper panels*) or presence of VEGF (*lower panels*) are shown on the *left hand panel*. Quantification of the network area as determined by the Incucyte Zoom Angiogenesis software is shown on the *right hand panel*. Values (*n* = 3) are means ± s.e.m, *p < 0.05 *versus* siScr minus VEGF, ^#^*p* < 0.01 *versus* siScr plus VEGF.

A key step in the directed migration and angiogenic sprouting of endothelial cells is their polarization, characterized by the formation of a leading edge and elaboration of filopodia orientated toward a gradient of chemoattractant angiogenic cytokine. Endothelial cell polarization was examined initially in a scratch wound assay in which endothelial cells migrate into the wound area of a cell monolayer. Following wounding, endothelial cells become polarized, indicated by the orientation of the juxtanuclear Golgi apparatus toward the wounded area. IQGAP1 knockdown strongly inhibited endothelial polarization in this assay (Fig. 6*A*).

We next tested the effect of IQGAP1 knockdown on the directed migration of endothelial cells in response to a VEGF gradient. IQGAP1 knockdown significantly inhibited VEGF-induced chemotaxis of endothelial cells, similar to the effects of p130Cas knockdown (Fig. 6*B*). We also examined the role of IQGAP1 and p130Cas in the angiogenic response to VEGF in an organotypic coculture angiogenesis assay. Targeted siRNAs to IQGAP1 and p130Cas significantly reduced the vascular network area generated in response to VEGF treatment for 7 days (Fig. 6*C* and supplemental Fig. S9).

##### Role of the p130Cas/IQGAP1 Association in VEGF Signaling

We next investigated how p130Cas mediates signal relay from VEGF to IQGAP1 and more distal signaling networks important for cell migration. Knocking down IQGAP1 by siRNA had no effect on VEGF stimulation of p130Cas tyrosine phosphorylation or phosphorylation of VEGFR2 (supplemental Fig. S10) indicating that tyrosine kinase pathways mediating p130Cas tyrosine phosphorylation are upstream and independent of the VEGF-dependent association between p130Cas and IQGAP1.

**Fig. 7.**
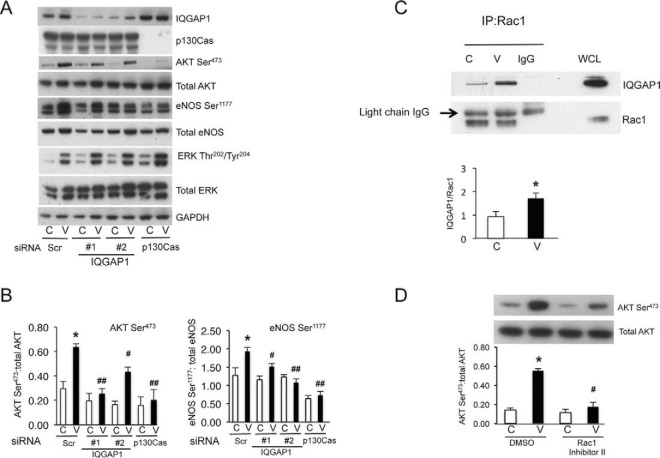
**Role of the p130Cas-IQGAP1 association in VEGF signaling.** *A*, HUVECS were transfected for 48 h with two different siRNAs targeting IQGAP1 (#1 and #2), p130Cas or siScr, all at a concentration of 200 nm. After incubation in EBM/0.5% FBS for 18 h, cells were stimulated with or without VEGF for 10 min, lysed and immunoblotted for the proteins indicated. Blots shown are representative of at least 3 independent experiments. *B*, Quantification of AKT Ser^473^ and eNOS Ser^1177^ phosphorylation; values (*n* = 3) are the AKTSer^473^:AKT and eNOSSer^1177^:eNOS ratios expressed as mean ± s.e.m. *p < 0.05 *versus* siScr minus VEGF, ^#^*p* < 0.05; ^##^*p* < 0.01 *versus* siScr plus VEGF. *C*, Association of IQGAP1 and Rac1 increases with VEGF stimulation. After incubation in EBM/0.5% FBS for 18 h, HUVECs were stimulated with or without VEGF for 10 min. Cells were then lysed and immunoprecipitated with anti-IQGAP1 antibody and immunoblotted for IQGAP1 and Rac1. Quantification of the immunoblots are shown below; values (*n* = 3) are the IQGAP1:Rac1 ratios expressed as mean ± s.e.m. *p < 0.05 *versus* vehicle control minus VEGF, by Student's t-Test. *D*, After incubation in EBM/0.5% FBS for 18 h, cells were incubated with 0.1 mm Rac1 Inhibitor II or 0.1% DMSO (vehicle control) for 4 h, followed by 10 min stimulation with or without VEGF. Cells were lysed and immunoblotted with antibodies to total and Ser^473^ phosphorylated Akt. Blots shown are representative of at least 3 independent experiments. Quantification of the immunoblots are shown below; values (*n* = 3) are the AKTSer^473^:AKT ratios expressed as mean ± s.e.m. *p < 0.05 *versus* vehicle control minus VEGF, ^#^*p* < 0.05 *versus* vehicle control plus VEGF.

The role of IQGAP1 in VEGF signaling was further examined by studying the effects of IQGAP1 knockdown on key VEGF signaling cascades important for endothelial cell migration. IQGAP1-targeted siRNA caused a significant inhibition of VEGF-induced Akt activation and eNOS phosphorylation at Ser^1177^, the major Akt-dependent eNOS phosphorylation site (Fig. 7*A*, 7*B*) (42). These findings suggested that the p130Cas interaction with IQGAP1 was important for Akt activation and downstream eNOS phosphorylation. This possibility was investigated by examining the effect of p130Cas-targeted siRNA on the Akt/eNOS pathway. Similar to the effects of IQGAP1 knockdown in cells cultured in parallel, p130Cas depletion strongly inhibited VEGF-induced Akt activation and eNOS phosphorylation at Ser^1177^ (Fig. 7*A*, 7*B*, supplemental Fig. S11). Another mechanism that could mediate the role of the IQGAP1 interaction in endothelial cell migration and angiogenesis is recruitment of the small GTPase Rac1, which has previously been implicated in the endothelial migratory response to VEGF. VEGF markedly increased the association between Rac1 and IQGAP1 as demonstrated by coimmunoprecipitation (Fig. 7*C*), consistent with previous findings (37). Preincubation of cells with a Rac1 inhibitor also significantly reduced VEGF-mediated AKT phosphorylation (Fig. 7*D*), supporting the conclusion that Rac1 mediates AKT activation downstream of IQGAP1. However, we could not demonstrate that p130Cas knockdown using two independent siRNAs significantly reduced VEGF-induced stimulation of IQGAP1 association with Rac1, suggesting that IQGAP1 may additionally modulate Rac1 activity and Rac1-mediated Akt activation via a p130Cas-independent pathway.

## DISCUSSION

Through its different domains, p130Cas is thought to function in the assembly of multiprotein signaling complexes required for remodeling of the actin cytoskeleton during cell motility (23). The *in vivo* functions of p130Cas are still poorly characterized, but the phenotype of p130Cas knockout mice demonstrates that this molecule is essential for mammalian embryonic cardiovascular development, p130Cas null mice display aberrant development of the heart, with thin myocardium, and severe dilatation of blood vessels, whereas other organs were largely unaffected (22). We have demonstrated that VEGF induces signaling via a NRP1-dependent p130Cas pathway that mediates endothelial cell chemotaxis (20). However, the mechanisms through which p130Cas regulates cell migration in endothelial cells are largely unknown.

A major conclusion from our analysis is that VEGF stimulates a major enrichment in the p130Cas interactome in proteins involved in actin-binding, the regulation of cell motility, and/or actin cytoskeletal dynamics. These included proteins previously found to play roles in cell migration, and/or angiogenesis and VEGF signaling, including IQGAP1, Profilin-1, MRCKβ, aldolase-A and formin homology domain proteins (FHOD or FMNL) 3. Thus VEGF-induced Profilin-1 phosphorylation is essential for post-natal angiogenesis in response to wounding and ischemia (43). The CDC42-binding kinase, MRCKβ, regulates actin organization in endothelial cell contacts and cell migration in cancer cells (44, 45), aldolase-A regulates lamellipodia formation (46), and FHOD3 is important for maintenance of F-actin polymerization at EC junctions and is required for vessel lumenization in angiogenesis (47). The presence of GTPase-regulating proteins in the p130Cas proteome indicates that p130Cas is an important node for relay of signals by VEGFRs to small GTPases important for endothelial migration and cellular morphogenesis. For example, VEGF also increased association of p130Cas with FDG5, an endothelial-specific protein which plays a key role in vessel pruning in mice in part through regulation of Cdc42 (48). VEGF also stimulated p130Cas association with other interactors of small GTPases, including MRCKβ, Slit-Robo Rho GTPase activating protein 1 (SRGAP1) and IQGAP1. Several proteins previously found to be associated with p130Cas were not identified by MS in the p130Cas interactome, most notably FAK and Crk. This may be because of the relatively low abundance of these interactions in endothelial cells, setting them below the threshold for detection by MS.

The VEGF-stimulated p130Cas interactome also contained several mRNA-binding proteins. Previous studies have drawn attention to FAs as sites of active, rapid mRNA and protein synthesis during integrin-dependent adhesion cells (49), an observation which likely reflects a role of actin-based structures and focal adhesions as scaffolds for polyribosome assembly in cells. Taken together, VEGF-induced enrichment in the p130Cas interactome of proteins involved in regulation of cell movement and adhesion, mRNA binding and stability, suggests an important role of p130Cas in integrating cell movement with the regulation of protein expression, which may be important for coordination of these two processes in the assembly and disassembly of actin filaments and FAs.

IQGAP1 was strongly associated with the VEGF-dependent p130Cas interactome, a finding that was confirmed independently by coimmunoprecipitation, by pull-down of p130Cas in endothelial cell lysates with recombinant IQGAP1, and by pull-down of IQGAP1 with recombinant p130Cas. IQGAP1 associated specifically with the amino-terminal p130Cas moiety containing the SH3, Proline-rich and kinase substrate domains. p130Cas tyrosine phosphorylation was required for its association with IQGAP1 in cells, because overexpression of a nontyrosine-phosphorylateable 15F p130Cas mutant prevented the association, whereas IQGAP1 was not necessary for VEGF-stimulated p130Cas tyrosine phosphorylation. Furthermore, VEGF-induced IQGAP1 association with phosphotyrosine was blocked by p130Cas knockdown, and by inhibition of Src, the main mediator of p130Cas tyrosine phosphorylation. Our findings do not demonstrate that IQGAP1 is itself directly phosphorylated by VEGF, as previous reports have suggested (41), but rather suggest that association of IQGAP1 with phoshotyrosine is more likely because of its association with p130Cas, a protein that is highly tyrosine phosphorylated in response to VEGF. Whether tyrosine phosphorylation of the p130Cas substrate domain is directly involved in IQGAP1 interaction is unclear. The IQGAP1 WW motif has the potential to associate with Proline-rich domains (38), raising the possibility that this interaction is with the p130Cas Proline-rich region. Further work will be necessary to precisely map the p130Cas-IQGAP1 interaction sites and the residues involved.

Our findings that IQGAP1 knockdown inhibits VEGF-induced endothelial cell polarization, migration, and angiogenesis, support the conclusion that VEGF induced association between p130Cas and IQGAP1 plays a key role in mediating VEGF chemotactic signaling. A role for p130Cas association with IQGAP1 in endothelial cell migration is also supported by the finding that VEGF markedly increased the concomitant recruitment of p130Cas and IQGAP1 to focal adhesions on fibronectin-adherent endothelial cells, whereas VEGF had no significant effect on FA association of paxillin, a major structural FA component and target of integrin signaling. These findings indicate an important role of p130Cas and IQGAP1 in VEGF-dependent signaling in FAs. A key mechanism that could mediate the role of the IQGAP1 interaction in endothelial cell migration and angiogenesis is Akt activation and eNOS ser^1177^ phosphorylation, a major Akt substrate (42). In support of this conclusion, we found that p130Cas and IQGAP1 were also both required for VEGF-induced Akt activation, and for eNOS ser^1177^ phosphorylation. Our findings suggest that IQGAP1 may also modulate Akt activation via an alternative pathway mediated via Rac1 that is independent of p130Cas.

This is the first analysis of the p130Cas interactome. Our finding that actin-binding and cytoskeletal-associated proteins represent a major class of p130Cas-interacting proteins is consonant with previous data indicating that p130Cas plays an important role in integrin-dependent signaling and cell motility. The identification of IQGAP1 as a major novel p130Cas binding partner and demonstration of a role for IQGAP1 in VEGF-dependent endothelial cell polarization and migration further reinforces the conclusion that the p130Cas interactome is a key component of the cell machinery important for cell migration, and plays a central role in the chemotactic response of endothelial cells to VEGF, with important roles in angiogenesis and in cardiovascular development. Determination of the endothelial role of p130Cas in explaining the lethal cardiovascular anomalies in p130Cas null mice will require analysis of conditional endothelial-specific p130Cas-deficient mice. Other findings from this analysis of the p130Cas interactome indicates previously unsuspected roles of p130Cas protein-protein interactions in linking the actin cytoskeleton to other cell functions such as regulation of mRNA stability. These results will be a basis for directing future studies elucidating the key interacting partners and mechanisms involved in mediating the functions of p130Cas and VEGF chemotactic and angiogenic signaling, and will also have wider implications for understanding mechanisms underlying cell movement and adhesion.

## Supplementary Material

Supplemental Data

## Footnotes

^1^ The abbreviations used are:

VEGF: vascular endothelial growth factor
VEGFR2: vascular endothelial growth factor receptor 2
PPI: protein-protein interactions
NRP1: neuropilin-1
PDGF: platelet derived growth factor
FAK: focal adhesion kinase
Ad: adenovirus
WT: wild type
HUVEC: human umbilical vein endothelial cell
MS: mass spectrometry
LFQ: label-free quantification
FDR: false discovery rate
GO: gene ontology.
