# Supplementary material for: Vascular Endothelial Growth Factor (VEGF) Promotes Assembly of the p130Cas Interactome to Drive Endothelial Chemotactic Signaling and Angiogenesis
Source: Mol Cell Proteomics. 2016 Dec 22;16(2):168–80. doi: 10.1074/mcp.M116.064428 (PMC5294206; doi:10.1074/mcp.M116.064428)
Supplement: Supplemental Data [file supp_16_2_168__index.html]

VEGF promotes assembly of the p130Cas interactome to drive endothelial chemotactic signalling and angiogenesis — Vascular Endothelial Growth Factor (VEGF) Promotes Assembly of the p130Cas Interactome to Drive Endothelial Chemotactic Signaling and Angiogenesis — VEGF-dependent Assembly of the p130Cas Interactome — Supplemental Data 

# Vascular Endothelial Growth Factor (VEGF) Promotes Assembly of the p130Cas Interactome to Drive Endothelial Chemotactic Signaling and Angiogenesis

## Supplemental Data

- Supplemental Figures (.pdf, 10.8 MB) - Supplementary methods and figures
- Supplemental Table 1 (.xlsx, 3.7 MB) - Supplemental Table 1
- Supplemental Table 2 (.xlsx, 9.7 MB) - Supplemental Table 2
- Supplemental Table 3 (.xlsx, 175 KB) - Supplemental Table 3
- Supplemental Table 4 (.xlsx, 434 KB) - Supplemental Table 4
- Supplemental Table 5 (.pdf, 109 KB) - Supplemental Table 5
- Supplemental Table 6 (.pdf, 72 KB) - Supplemental Table 6
