# Supplementary material for: Vascular Endothelial Growth Factor (VEGF) Promotes Assembly of the p130Cas Interactome to Drive Endothelial Chemotactic Signaling and Angiogenesis
Source: Mol Cell Proteomics. 2016 Dec 22;16(2):168–80. doi: 10.1074/mcp.M116.064428 (PMC5294206; doi:10.1074/mcp.M116.064428)
Supplement: Supplemental Data [file 10.1074_M116.064428_mcp.M116.064428-1.pdf]

Supplementary material

Materials and Methods

Cell culture

Human umbilical vein endothelial cells (HUVECs) were purchased from TCS CellWorks (Buckingham, UK) and cultured in endothelial basal medium, (EBM; Cambrex BioScience Ltd, Nottingham, UK) supplemented with gentamycin-ampicillin, epidermal growth factor and bovine brain extract (Singlequots; Cambrex) and 10% FCS. HUVECs used in experiments were no more than passage 6.

Adenoviral construction and infection

Adenoviruses (Ad) expressing p130Cas, and p130Cas15F were generated as previously described[20]. HUVECs were infected with Ad expressing either LacZ (Ad.LacZ), p130Cas (Ad.p130Cas), or p130Cas15F (Ad. p130Cas15F) at an MOI of 250.

Antibodies, reagents and siRNA

Antibodies against phospho-p130Cas (Y410), ERK, phospho-ERK (T202/Y204), AKT, phospho AKT (S473), KDR, phospho-KDR (Y1175) and phospho-tyrosine were from Cell Signaling Technology (Danvers, MA). Antibodies against GAPDH (V-18), p130Cas (rabbit polyclonal), neuropilin (C-19), paxillin and secondary antibodies against mouse, goat and rabbit IgGs were from Santa Cruz Biotechnology (Santa Cruz, CA). Antibodies to p130Cas (mouse monoclonal) and GM130 were from BD Transduction Laboratories (Oxford, UK). Anti-vinculin antibody and TRITC-phalloidin were from Sigma (Dorset, UK) Alexa-Fluor-486-conjugated donkey anti-goat-IgG, Alexa-Fluor-546-conjugated donkey anti-rabbit-IgG and Alexa-Fluor-555–phalloidin were from Life Technologies (Paisley, UK). VEGF-A_{165} was purchased from Peprotech (London, UK).

The siRNAs used are detailed below:

siRNA	Sequence (5’- 3’)	Supplier
IQGAP1#1	CAATGATCCAATCCACGAAtt	Life Technologies
IQGAP1#2	GCAGGTGGATTACTATAAA	GE Healthcare
NRP1#1	CGACAGCGCGAUAGCAAAAtt	Life Technologies

NRP1#2	GGAUUUUCCAUACGUUAUGtt	Life Technologies
p130Cas#1	GAGTTTGAGAAGACCCAGAtt	Life Technologies
p130Cas#2	GGUCGACAGUGGUGUGUAU	GE Healthcare

Immunofluorescent staining and confocal imaging

For immunofluorescent staining, cells were fixed in 4% paraformaldehyde in PBS for 1 hour followed by permeabilisation in 0.2% Triton X-100 for 30 minutes. After blocking in 10% bovine serum albumin (BSA) in PBS for 1 hour, antibody incubations were performed overnight at 4°C. Confocal imaging was performed using a LEICA SPE2 upright microscope running LEICA-LAS software using sequential imaging capture.

Focal adhesion isolation

Focal adhesion isolation was performed as described(36). Briefly, cells were washed with PBS and incubated with TEA buffer (0.2 M triethanolamine, pH 8.0) for 5 min. To apply hydrodynamic force, the cells were rinsed with PBS for 10 s using a Waterpik dental flosser set at 2 (Waterpik, Reigate, UK). After another wash with PBS, the remaining attached focal adhesions were either fixed for immunofluorescent staining, or were lysed for western blot analysis.

siRNA transfection

HUVECs at 70% confluence were transfected with Oligofectamine Reagent (Invitrogen) and 200 nM siRNA as described previously(20).

Transwell chemotactic migration assay

This assay was performed as described previously(20). Briefly, transwell cell culture inserts (Falcon; BD Biosciences, Oxford, UK) were inserted into a 24-well plate. Serum-free medium (SFM) with or without the indicated growth factors or the vehicle was placed in the bottom chamber, and cells in suspension (1.5 x10^5/well in serum-free EBM were added to the top chamber and incubated at 37°C for 4 h. Cells that had not migrated or had only adhered to the upper side of the membrane were removed before the membrane was fixed and stained with a Reastain Quik-Diff kit (IBG Immucor Ltd., West Sussex, UK) using the manufacturer's protocol and mounted on a glass slide. Cells that had migrated to the lower side of the membrane were counted in

four random fields per well at x20 magnification using an indexed eyepiece graticule.

Co-Culture angiogenesis assay

Human dermal fibroblasts were grown to confluence in a 24 well plate. HUVECs were trypsinised and plated onto the fibroblasts (1 x10^4 cells/well) in EBM plus 1% FBS. Cells were stimulated with or without VEGF (25 ng/ml) and cultured for seven days with a media change at day four. After seven days, cells were fixed in ethanol and endothelial cells were detected by probing with an anti von Willebrand factor antibody followed by a secondary antibody conjugated with Alexa Fluor 488. Cells were visualised using an Incucyte Zoom (Essen Bioscience) and parameters of tube formation were determined using the Incucyte Zoom Angiogenesis software package.

Recombinant protein generation and Pulldown

The pGex-2T-IQGAP1 construct (Addgene; Cambridge, MA) was transformed into BL21 (D3) cells for protein expression. Expression of the GST fusion protein was induced overnight at 24°C in the presence of 0.1mM IPTG. Bacteria (from 100ml culture) were pelleted, resuspended in lysis buffer containing 50mM Tris-HCl at pH8, 300 mM NaCl, 1mM EDTA, 3mM DTT, 0.1% Triton X100 and lysozyme, then sonicated. Insoluble material was pelleted at 11,000xg for 15 min and 200 μl of a 50% slurry of Glutathione Sepharose 4B beads (GE Healthcare) was added to the clarified supernatants. The beads were allowed to bind proteins for 1 hour, washed 3x in PBS and finally resuspended in 150ul of PBS. Protein expression was assessed by Coomassie staining of SDS-PAGE gels. SDS-PAGE gels were rinsed once in dH_2O and then 20 ml of staining solution (40% methanol, 10% acetic acid, 0.1% Coomassie Blue) was added for 2 hours. Gels were then rinsed twice in dH_2O and exposed to destaining solution overnight (5% Methanol, 7.5% acetic acid).

p130Cas mutants containing (residues 1-433: F1) or (residues 431-888; F2) were generated by PCR using specific primers. PCR product were then digested by BamH1 and Xho1 restriction enzymes, and cloned into pGex-4T-2 vector in frame with GST coding sequences. Constructs were then transformed into BL21 cells for protein expression. Expression of GST fusion proteins was induced by 1mM IPTG at 24°C overnight. Bacteria were pelleted, resuspended in lysis buffer containing 50 mM Tris-HCl at pH8, 300 mM NaCl, 1mM EDTA, 3 mM DTT and 0.1% Triton X100,

then sonicated. Fusion proteins were recovered by incubation with glutathione-agarose beads overnight at 4ºC with mixing, subsequently washed three times with PBS and supplemented with SDS sample buffer.

Pulldown experiments were performed by incubating GST-fusion proteins with HUVEC cell lysates overnight 4ºC, recovering GST-fusions with glutathione—conjugated agarose beads, subsequently washing three times with ice cold PBS, and extracting protein with SDS sample buffer. Samples were separated by gel electrophoresis, transferred and immunoblotted as indicated.

Primers:

F1_F
CATGGGATCCATGAACCACCTGAACGTGCTGG

F1_R
CGCTCGAGCTATGGGGGAGGCACCGCATACA

F2_F
CATGGGATCCCCTCCCCCAGCTGAACGTGAAG

F2_R
CGCTCGAGTCAGGCGGCTGCCAGCTGGCCTA

Legends to Supplementary tables

Table S1. MaxQuant output ‘proteinGroups.txt’ file containing identified proteins, m/z values, number of peptides and % sequence coverage following removal of reverse and contaminant ids.

Table S2. MaxQuant output ‘peptides.txt’ file containing peptide sequences, peptide identification score and charge state.

Table S3. BDiffProt output for the analysis of the previously published AP-MS data set.

Table S4. BDiffProt output including fold changes for each condition and proteins excluded from further analysis. WT INT and MT INT indicates wild type and mutant interactors respectively. The proteins which have "NO" in both columns were not considered for further analysis.

Table S5. List of proteins identified in the p130Cas WT interactome, subdivided into functional categories. Y indicates significant interaction with p130Cas at that time point

Table S6. List of proteins identified in the p130Cas 15F interactome, subdivided into functional categories. Y indicates significant interaction with p130Cas at that time point

Legends to Supplementary figures

Figure S1. Venn diagram based comparison of the results of BDiffProt analysis and the published list of differentially interacting proteins. The BDiffProt algorithm was used to analyse a previously published AP-MS data set(33). The Venn diagram shows the number of proteins identified as being differentially expressed using BDiffProt (Blue circle), or as determined in the original publication (pink circle). The number of proteins commonly identified in both analyses is shown in the overlapping magenta area.

Figure S2. P130Cas input into the Mass Spectrometry. The amount of p130Cas from each of the IP replicates was analysed and is shown on the graph as the log intensity values as measured by the Mass Spectrometer.

Figure S3. Endogenous p130Cas association with profilin1, and MRCKβ. Cells were incubated in EBM/0.5% serum for 18 hours prior to treatment with VEGF for the times indicated. Cells were lysed and immunoprecipitated with anti-p130Cas antibody and immunoblotted with the indicated antibodies. Images are representative of at least 2 independent experiments.

Figure S4. IQGAP1 associates with tyrosine phosphorylated protein(s). Cells were transfected with 200 nM sip130Cas or 200 nM of siScr, for 48h. After overnight incubation in EBM/0.5% FBS, cells were stimulated with or without VEGF for 15 minutes. Cells were lysed and immunoprecipitated with anti-phosphotyrosine

antibody and immunoblotted with an antibody to IQGAP1. Quantification of the co-immunoprecipitation data by densitometry using ImageJ is shown below. *P < 0.05 vs siScr minus VEGF, #P < 0.01 vs siScr plus VEGF, n=3. (**B**) After overnight incubation in EBM/0.5% FBS, cells were preincubated for 30 minutes with either DMSO (0.1%), 10 μM PP2 (Src family kinase inhibitor) or 5 μM PF228 (FAK inhibitor) followed by 15 minutes stimulation with or without VEGF. Cells were lysed and immunoprecipitated with anti-phosphotyrosine antibody and immunoblotted with an antibody to IQGAP1. In addition, WCLs were probed with the indicated antibodies. A blot representative of 3 independent experiments is shown, the dashed line between the phospho tyrosine IP samples and WCL indicates that the WCL lane was originally on another part of the same gel. Quantification of IQGAP1 tyrosine phosphorylation is shown below. Values (n=3) are expressed as mean ± s.e.m. *P < 0.05 vs DMSO minus VEGF, #P< 0.01 vs DMSO plus VEGF.

Figure S5. IQGAP1 associates with the N-terminal region of p130Cas. pGex-2T-IQGAP1 construct were transformed into BL21 (D3) cells for protein expression. Expression of GST fusion protein was induced overnight at 24°C in the presence of 0.1mM IPTG. Bacteria were pelleted, resuspended in lysis buffer, then sonicated. Insoluble materials were pelleted and a 50% slurry of Glutathione Sepharose 4B beads was added to the clarified supernatants. The beads were allowed to bind proteins for 1h, washed 3x in PBS and finally resuspended in of PBS. (**A**) GST-IQGAP1 or GST-alone fusion proteins were separated by SDS PAGE and stained with Coomassie Blue. (**B**) GST-IQGAP1 or GST alone fusion proteins were incubated with cell lysates overnight 4°C, subsequently washed three times with ice cold PBS and supplemented with SDS sample buffer. Samples were separated by gel electrophoresis, transferred and immunoblotted with anti-p130Cas antibody. (**C**) N-terminal (residues 1-433) and C-terminal (residues 431-888) p130Cas mutants were generated by PCR using specific primers as detailed in Materials and Methods. Constructs were then transformed into BL21 cells for protein expression. Expression of GST fusion proteins was induced as detailed above. GST-N-terminal p130Cas, GST-C-terminal p130Cas or GST alone fusion proteins were incubated with cell lysates overnight at 4°C, subsequently washed three times with ice cold PBS and

supplemented with SDS sample buffer. Samples were separated by gel electrophoresis, transferred and immunoblotted with anti-IQGAP1 antibody.

Figure S6. Requirement for NRP1 in IQGAP1-p130Cas association. Cells were transfected with 200 nM siNRP1#2 or 200 nM siScr for 48h, incubated in EBM/0.5% serum for a further 18 hours, then stimulated with or without VEGF for 30 minutes, lysed and immunoprecipitated with anti-p130Cas antibody and immunoblotted with antibodies to p130Cas and IQGAP1. Quantification of co-immunoprecipitated IQGAP1 is shown below. $^{\#}P < 0.05$ versus siScr minus VEGF, $^{*}P < 0.01$ vs siScr plus VEGF, n=3.

Figure S7. VEGF induces IQGAP1 translocation to focal adhesions. HUVECs were plated onto coverslips and after incubation in low serum (0.5%) EBM for 18 hours, cells were stimulated with or without VEGF for 30 min. Cells were fixed with 4% PFA and then: **A**, stained using Phalloidin-TRITC (red; F-actin), and co-stained using antibodies to IQGAP1 (green), or p130Cas (green), and nuclei were visualised using DAPI (blue); or **B**, stained using antibodies to IQGAP1 (green), and Vinculin (red), and nuclei were visualised using DAPI (blue). Shown are representative images from six different slides. The zoomed areas show increased colocalization between vinculin and IQGAP1 after VEGF treatment.

Figure S8. Paxillin staining in intact cells and after focal adhesion isolation. Immunofluorescent staining of intact cells and isolated focal adhesions using the nuclear stain DAPI (blue) and an antibody to paxillin (green). DAPI is entirely absent from the isolated focal adhesions, confirming the removal of nucleus. In addition, paxillin staining is limited to focal adhesions after isolation whereas some cytoplasmic staining is observed in intact cells.

Figure S9. Requirement for p130Cas in VEGF-induced angiogenesis. HUVECS were transfected with 200 nM siRNA targeting p130Cas (p130Cas#2) or siScr for 48 h. Cells were trypsinised and 1 $x10^4$ cells were applied to a confluent monolayer of human dermal fibroblasts in EBM supplemented with 1% FBS and with or without 25 ng/ml VEGF. Co-cultures were incubated for 7 days, with a change of media after 4

days, before being fixed in ethanol. Endothelial cells were stained with anti-VWF antibody followed by a biotinylated secondary antibody (Chemicon) and ABC (Vector Laboratories Inc.) with DAB staining (Sigma). Photomicrographs of VWF-stained co-cultures were analyzed using ImageJ software. The length of all tubular structures (stained endothelial cells) and the number of branching points were measured in four representative microscopic fields per well. Values (n = 3) are means ± s.e.m, *P < 0.05 vs siScr minus VEGF, $^{\#}$P< 0.01 vs siScr plus VEGF.

Figure S10. IQGAP1 knockdown does not alter VEGF-induced KDR or p130Cas phosphorylation. HUVECS were transfected with two different siRNAs targeting IQGAP1 at a concentration of 200 nM, or 200 nM of siScr. After incubation in low serum (0.5%) EBM for 18 hours, cells were stimulated with or without VEGF for 10 min cells were lysed and immunoblotted for the proteins indicated.

Figure S11. Requirement for p130Cas in VEGF-induced Akt and eNOS phosphorylation. HUVECS were transfected for 48 h with siRNAs targeting p130Cas (p130Cas#2) or siScr, at a concentration of 200 nM. After incubation in EBM/0.5% FBS for 18 hours, cells were stimulated with or without VEGF for 10 min, lysed and immunoblotted for the proteins indicated. Blots shown are representative of at least 3 independent experiments. (**B**) Quantification of AKT S473 and eNOS S1177 phosphorylation; values (n=3) are the AKTpS473:AKT and eNOSpS1177:eNOS ratios expressed as mean ± s.e.m. *P < 0.05 vs siScr minus VEGF, $^{\#}$P< 0.05; $^{\#\#}$P< 0.01 vs siScr plus VEGF.

Suppl Fig. 1

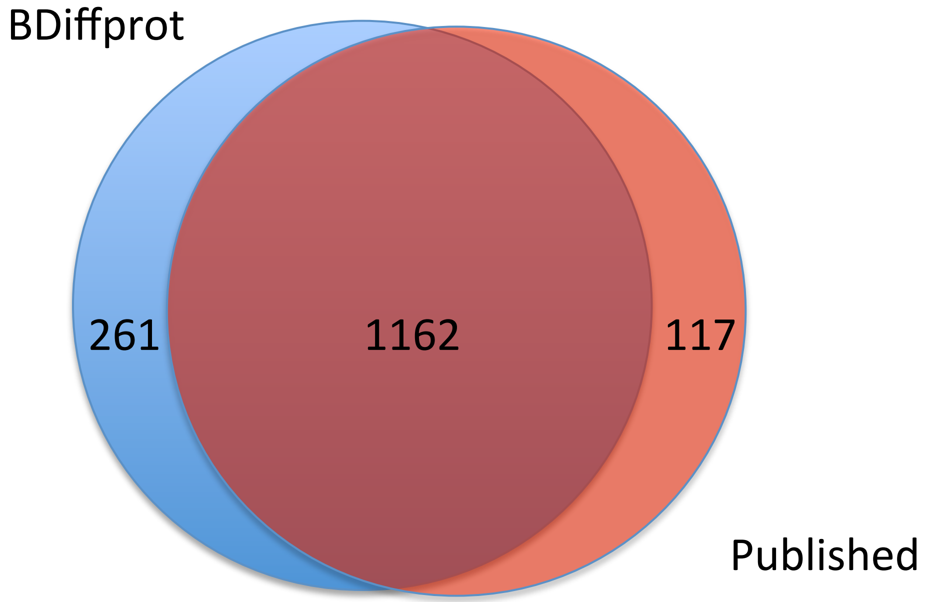

Suppl Fig. 2

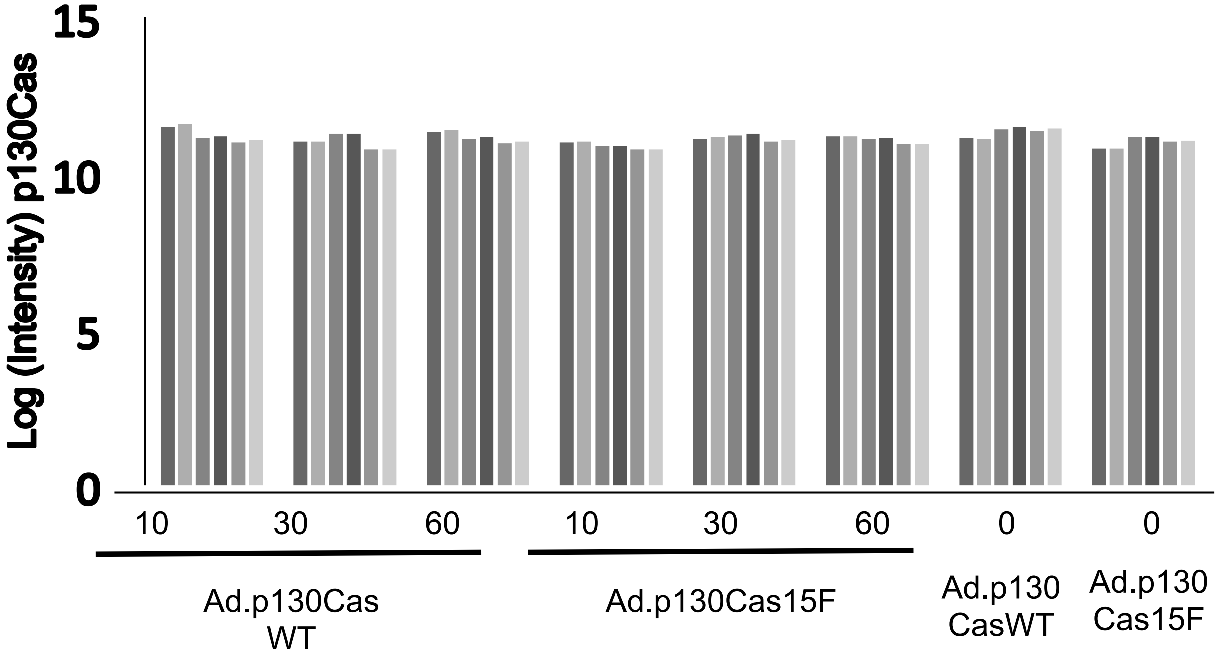

Log (Intensity) p130Cas
15
10
5
0
10
30
60
10
30
60
0
0
Ad.p130Cas WT
Ad.p130Cas15F
Ad.p130 CasWT
Ad.p130 Cas15F

Suppl Fig. 3

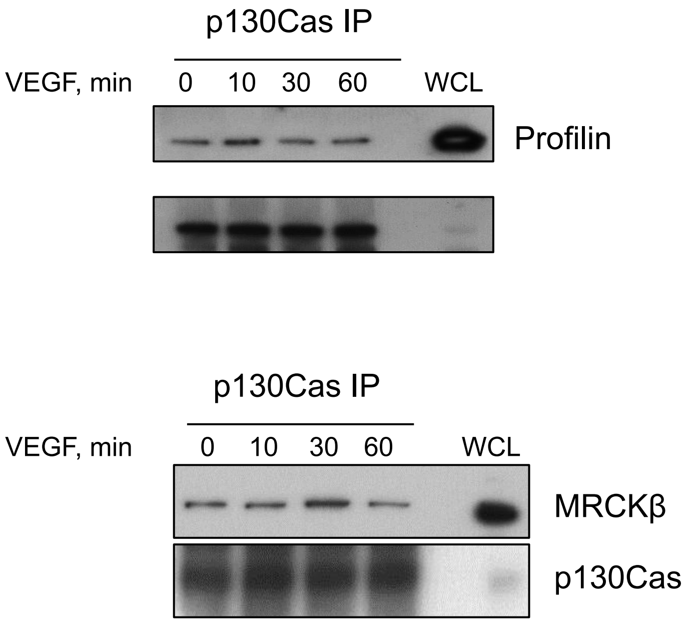
p130Cas IP
VEGF, min
0
10
30
60
WCL
Profilin
p130Cas IP
VEGF, min
0
10
30
60
WCL
MRCKβ
p130Cas

Suppl Fig. 4

A

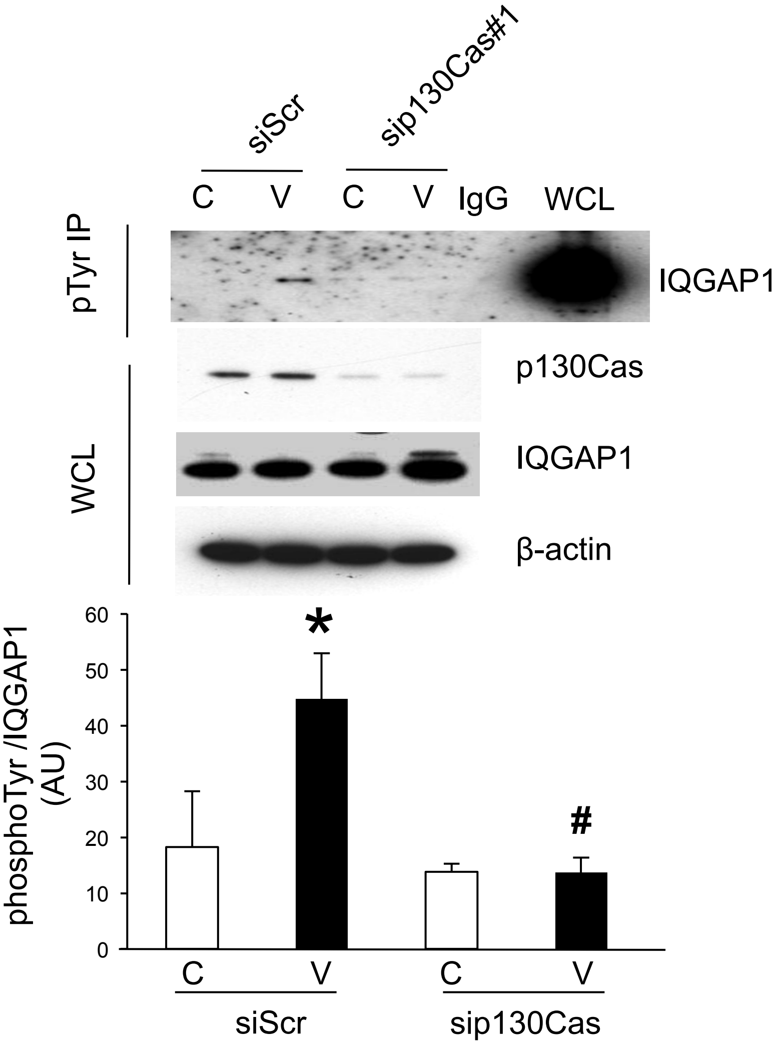

B

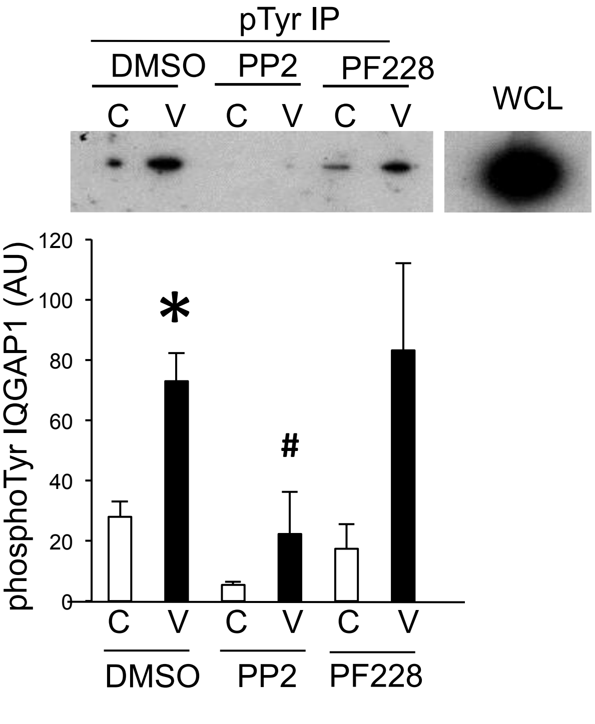

Suppl Fig. 5

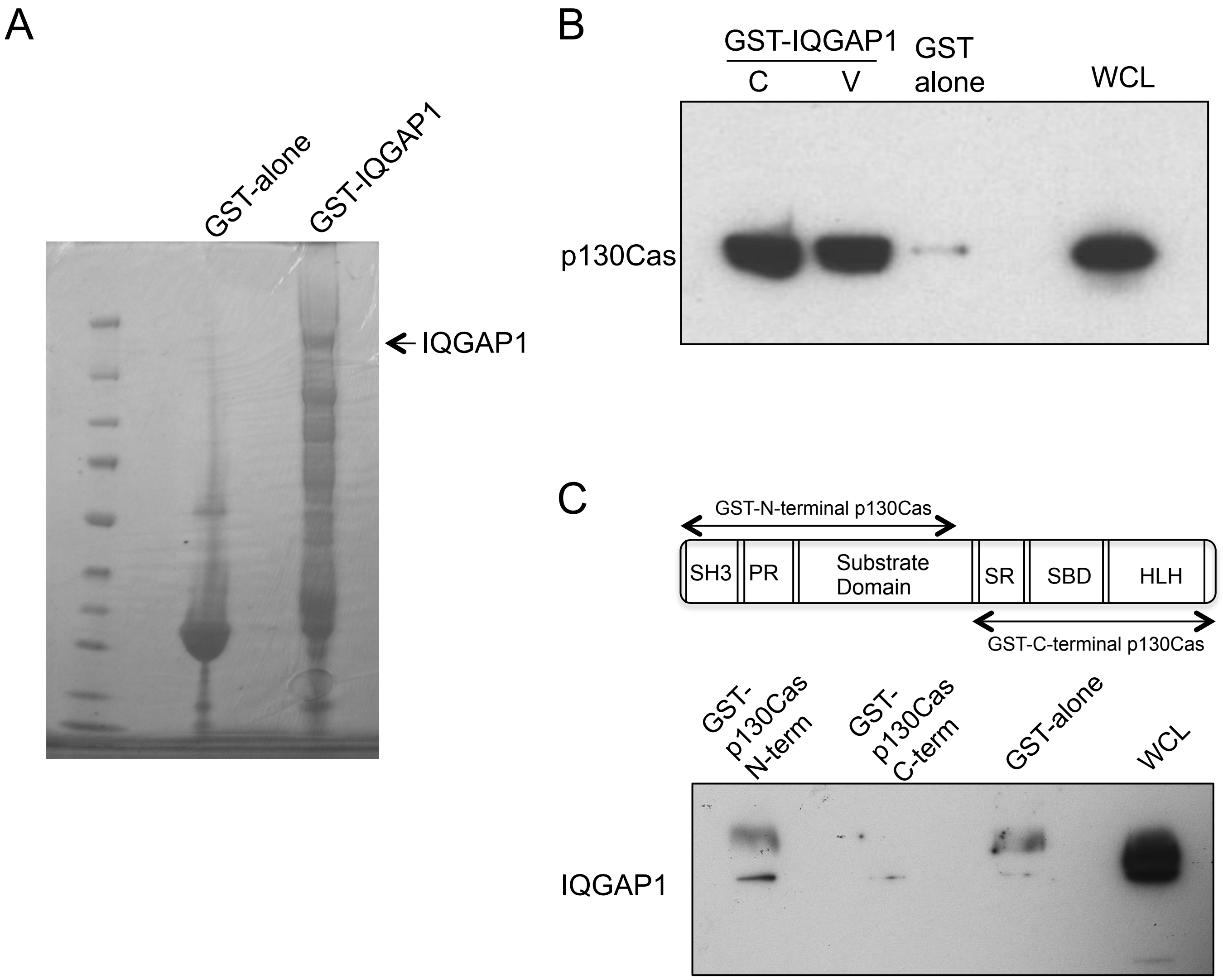

A
GST-alone
GST-IQGAP1
IQGAP1
B
GST-IQGAP1
C
V
GST alone
WCL
p130Cas
C
GST-N-terminal p130Cas
SH3
PR
Substrate Domain
SR
SBD
HLH
GST-C-terminal p130Cas
GST-p130Cas N-term
GST-p130Cas C-term
GST-alone
WCL
IQGAP1

Suppl Fig. 6

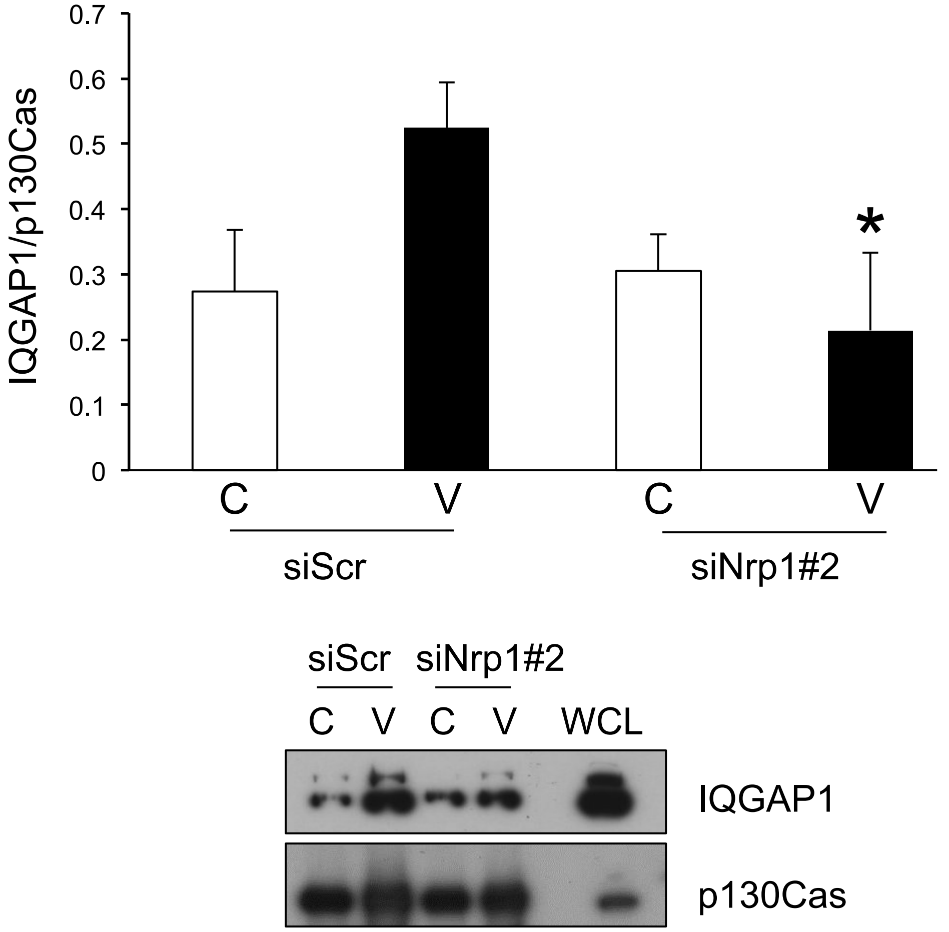

IQGAP1/p130Cas
0.7
0.6
0.5
0.4
0.3
0.2
0.1
0
*
C
V
C
V
siScr
siNrp1#2
siScr
siNrp1#2
C
V
C
V
WCL
IQGAP1
p130Cas

Suppl Fig. 7A

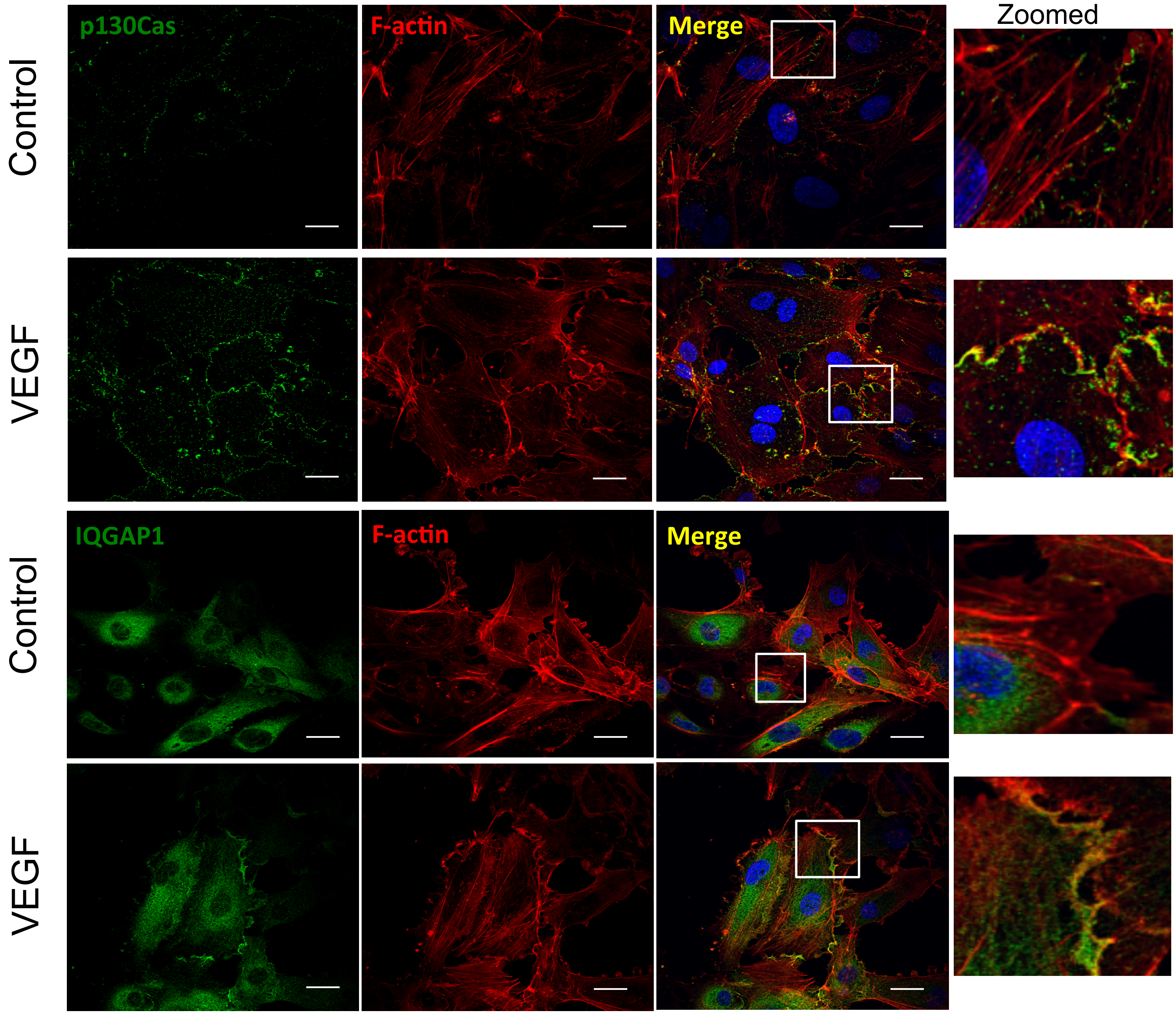
Zoomed
Control
VEGF
Control
VEGF
p130Cas
F-actin
Merge
IQGAP1
F-actin
Merge

Suppl Fig. 7B

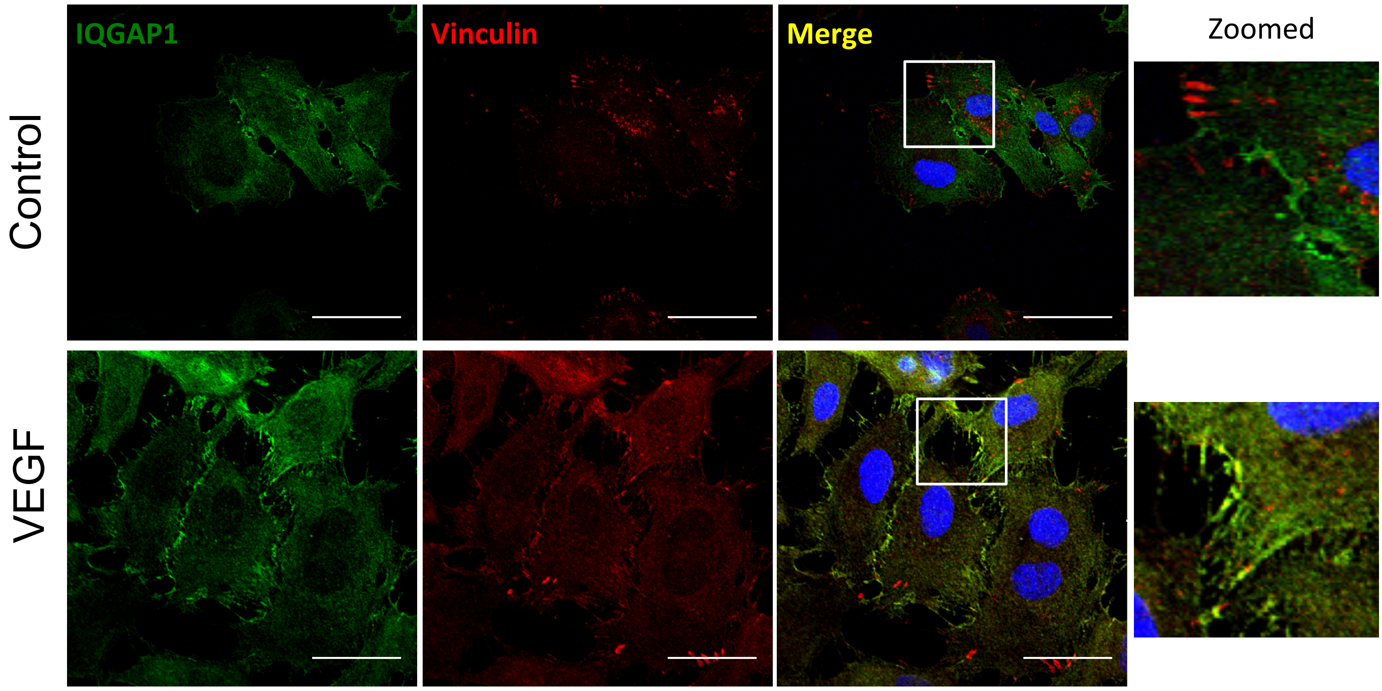

Suppl Fig. 8

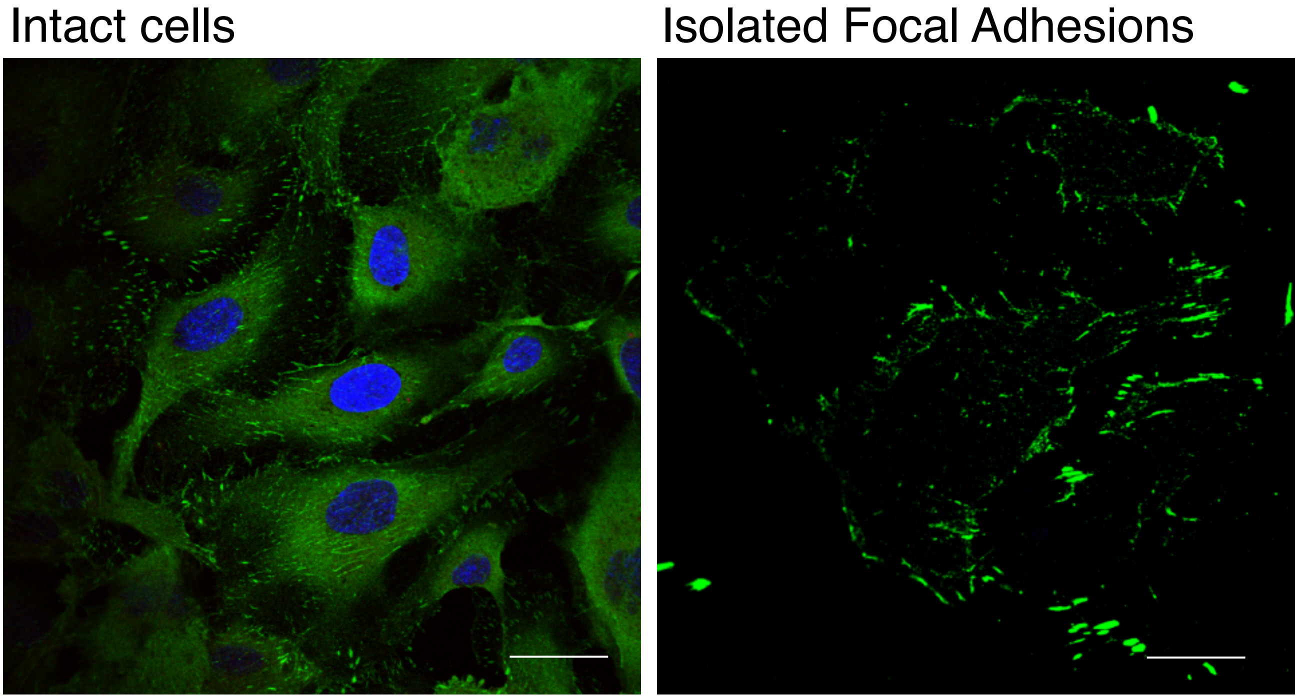

Paxillin – green DAPI - Blue

Suppl Fig. 9

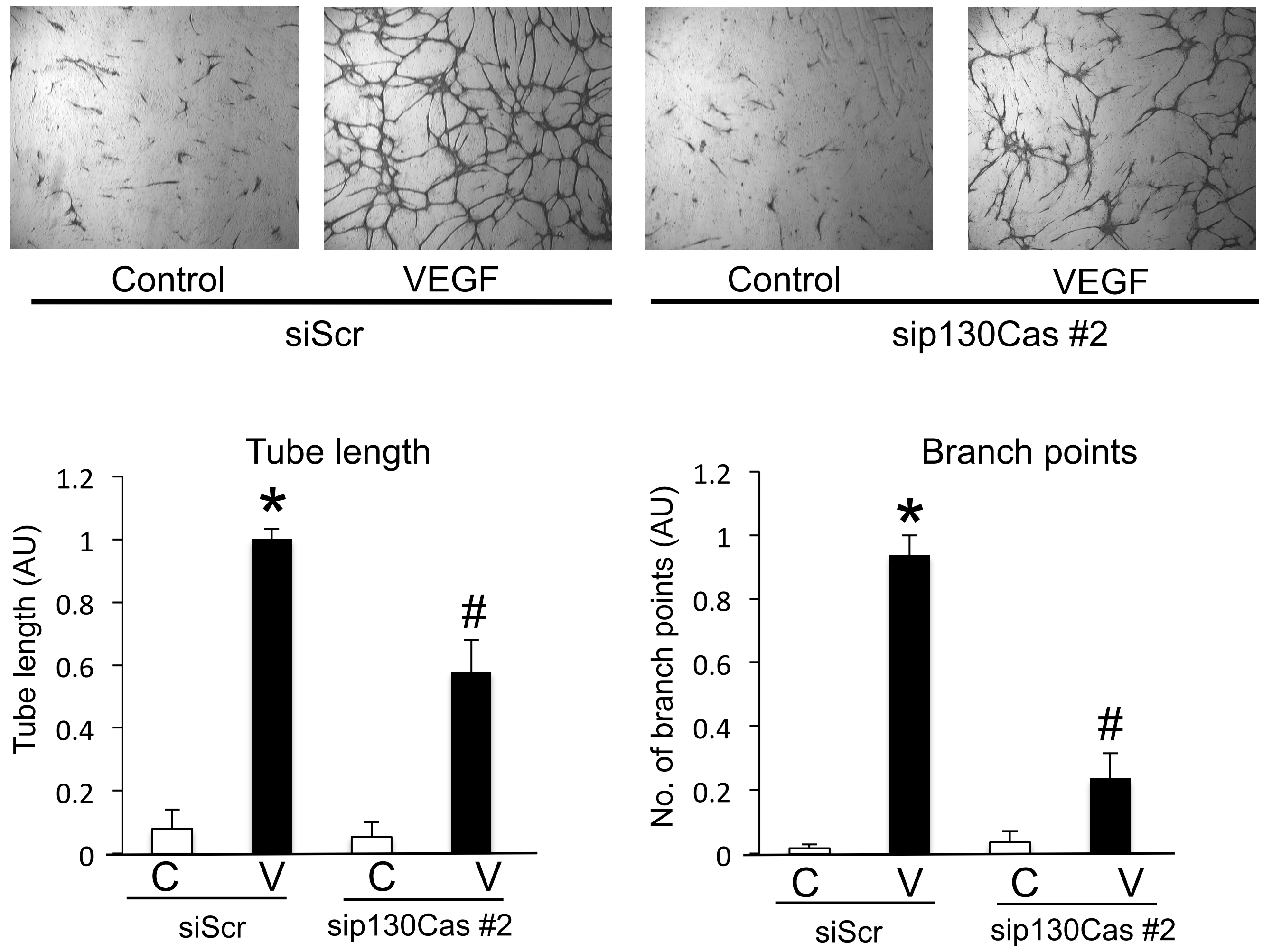
Control
VEGF
siScr
Control
VEGF
sip130Cas #2
Tube length
Tube length (AU)
1.2
1
0.8
0.6
0.4
0.2
0
*
#
C
V
C
V
siScr
sip130Cas #2
Branch points
No. of branch points (AU)
1.2
1
0.8
0.6
0.4
0.2
0
*
#
C
V
C
V
siScr
sip130Cas #2

Suppl Fig. 10

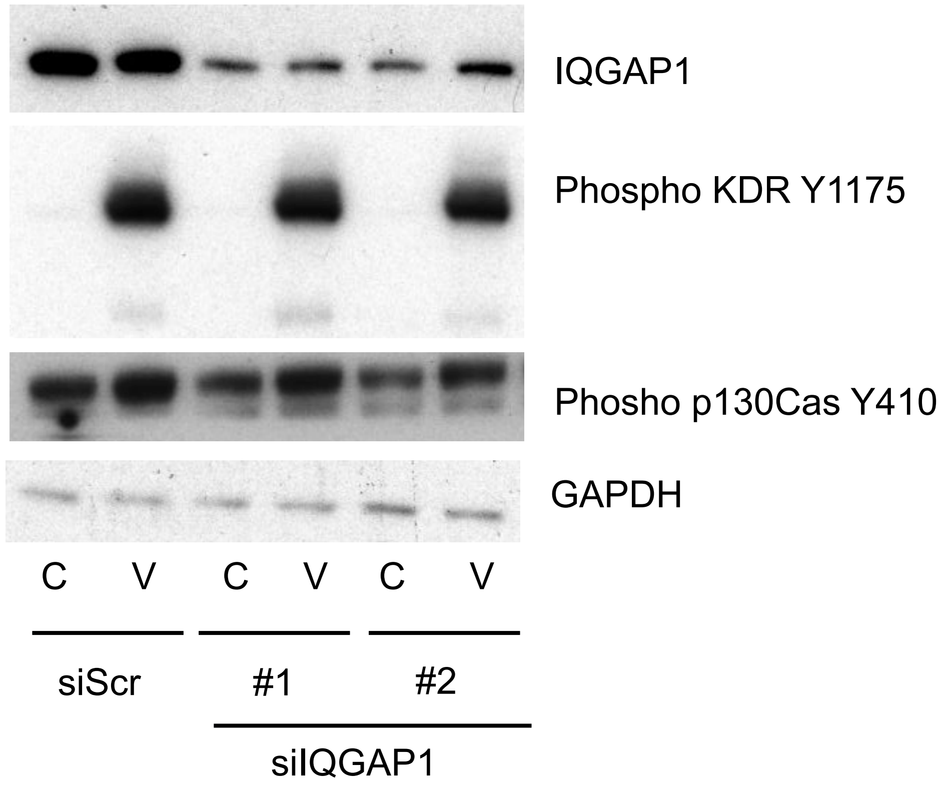
IQGAP1
Phospho KDR Y1175
Phosho p130Cas Y410
GAPDH
C V C V C V
siScr
#1
#2
siIQGAP1

Suppl Fig. 11

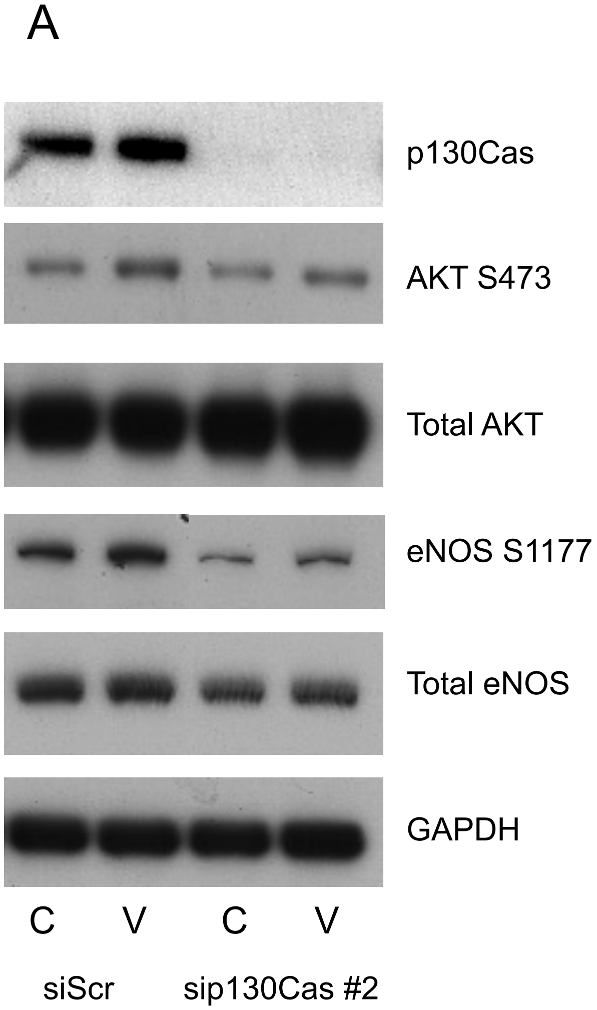
A
p130Cas
AKT S473
Total AKT
eNOS S1177
Total eNOS
GAPDH
C V C V
siScr
sip130Cas #2

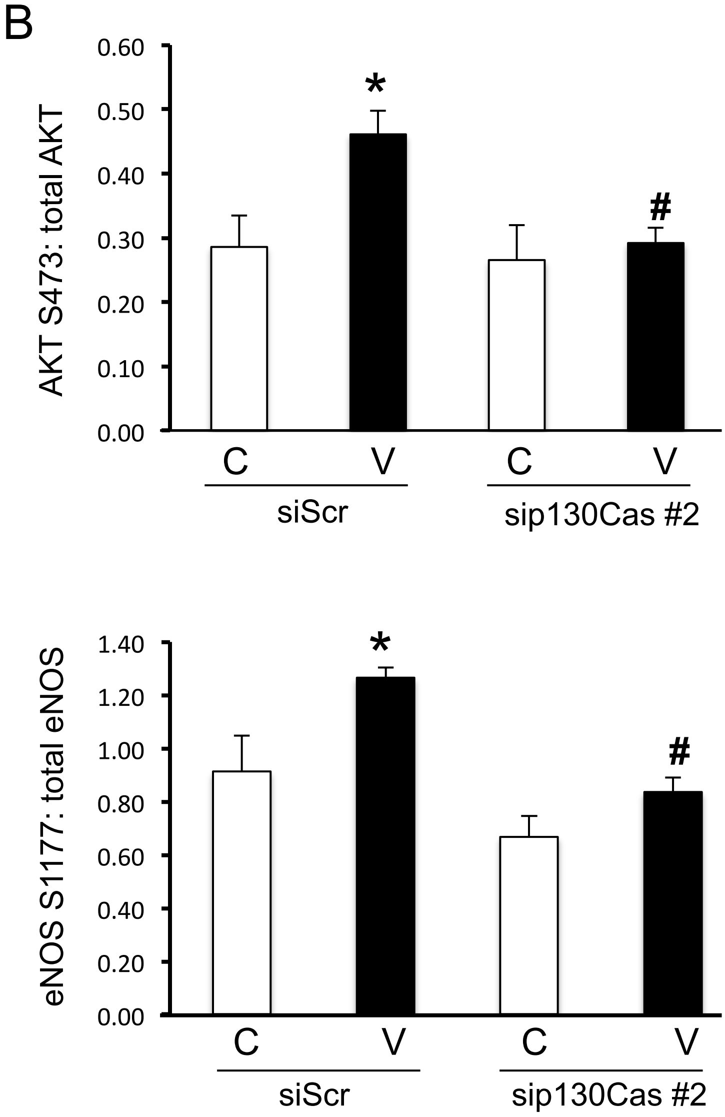
B
AKT S473: total AKT
0.60
0.50
0.40
0.30
0.20
0.10
0.00
*
#
C V C V
siScr
sip130Cas #2
eNOS S1177: total eNOS
1.40
1.20
1.00
0.80
0.60
0.40
0.20
0.00
*
#
C V C V
siScr
sip130Cas #2
