# Supplementary material for: Vascular Endothelial Growth Factor (VEGF) Promotes Assembly of the p130Cas Interactome to Drive Endothelial Chemotactic Signaling and Angiogenesis
Source: Mol Cell Proteomics. 2016 Dec 22;16(2):168–80. doi: 10.1074/mcp.M116.064428 (PMC5294206; doi:10.1074/mcp.M116.064428)
Supplement: Supplemental Data [file 10.1074_M116.064428_mcp.M116.064428-6.pdf]

Gene Symbol	Name	Function	VEGF, min 10	30	60
**Cytoskeleton**					
KATNAL2	Katanin p60 subunit A like 2	ATPase activity			Y
KIF1C;KIF1A;KIF1B	Kinesin family member 1	Motor activity		Y	Y
MYH11	Myosin heavy chain, smooth muscle isoform	Structural molecule activity	Y	Y	Y
MYO1C	Myosin IC	Motor activity			Y
MYO6	Myosin 6	Motor activity	Y		Y
TBCA	Tubulin-specific chaperone a	Chaperone activity	Y		Y
TMOD1	Tropomodulin	Cytoskeletal protein binding		Y	
TMOD3	Tropomodulin 3	Cytoskeletal protein binding	Y	Y	Y
TPM3	Tropomyosin 3	Cytoskeletal protein binding		Y	Y
TUBA1C	Tubulin alpha 6	Structural constituent of cytoskeleton	Y		Y
TUBB8	Tubulin, beta 8	Structural molecule activity			Y
**Actin cytoskeleton**					
ACTG1	Actin gamma 1	Structural constituent of cytoskeleton	Y	Y	Y
ACTN1	Alpha-actinin-1	Cytoskeletal binding protein			Y
ARPC5L	Actin related protein 2/3 complex subunit 5 like	Cytoskeletal binding protein	Y		Y
CAPZB	F actin capping protein beta subunit	Structural constituent of cytoskeleton		Y	
EPB41	Erythrocyte membrane protein band 4.1	Structural molecule activity		Y	Y
FHOD1	Formin homology 2 domain containing 1	Cytoskeletal binding protein			Y

FLII	Flightless 1	Cytoskeletal binding protein			Y
FMNL3	Formin like 3	Actin binding protein	Y	Y	Y
FSCN1	Fascin 1	Structural molecule activity			Y
IQGAP1	IQ motif containing GTPase activating protein 1	GTPase activator activity		Y	Y
KANK1	Ankyrin repeat domain 15	Rho activity regulation			Y
MICAL2	Flavoprotein oxidoreductase MICAL2	Cytoskeletal protein binding		Y	Y
NEXN	Nexilin	Cytoskeletal protein binding			Y
PFN1	Profilin 1	Cytoskeletal protein binding	Y	Y	Y
SHROOM2	Shroom family member 2	Actin binding protein			Y
SPIRE1	Spir1 protein	Transporter activity			Y
TMSB4X;TMSB4XP4	Thymosin, beta-4	Cytoskeletal protein binding	Y		Y
**Cell Migration**					
CDC42BPB	CDC42 binding protein kinase beta	Protein serine/threonine kinase activity	Y	Y	
CGNL1	Cingulin-like 1	Rho GTPase activity regulation			Y
CRIP2	Cystein rich intestinal protein 2	Receptor signaling complex scaffold activity			Y
CSRP1	Cysteine and glycine rich protein 1	Receptor signaling complex scaffold activity	Y		Y
CSRP2	Cysteine and glycine rich protein 2	Receptor signaling complex scaffold activity			Y
DSC1	Desmocollin 1	Cell adhesion molecule activity			Y
MARCKSL1	MARCKS like protein	Receptor signaling complex scaffold activity	Y		Y
MLLT4	Mixed lineage leukemia, translocated to 4	Cell adhesion molecule activity			Y
RDX	Radixin	Cytoskeletal protein binding			Y
SLFN5	Schlafen family member 5	Unknown			Y
SRGAP1	SLIT ROBO Rho GTPase activating	GTPase activator activity	Y		Y

	protein 1				
YWHAE	14-3-3 epsilon	Receptor signaling complex scaffold activity	Y	Y	Y
**Angiogenesis**					
FGD5	FYVE, RhoGEF and PH domain containing 5	Guanyl-nucleotide exchange factor activity	Y	Y	Y
PTPN14	Protein tyrosine phosphatase, non receptor, type 14	Protein tyrosine phosphatase activity			Y
TMSB10	Thymosin beta 10	Cytoskeletal protein binding	Y	Y	Y
**Protein Trafficking**					
AP2A1	Adaptor related protein complex 2 alpha 1 subunit	Transporter activity			
AP2S1	Clathrin adaptor complex AP2, sigma subunit	Receptor signaling complex scaffold activity	Y	Y	Y
ARF4	ADP ribosylation factor 4	Transporter activity	Y	Y	Y
NAPA;NAPB	Soluble NSF attachment protein	Receptor signaling complex scaffold activity	Y		
NUMB	Numb homolog	Clathrin-associated sorting			Y
UBAP2L	Ubiquitin associated protein 2 like	Ubiquitin associated protein			Y
VPS39	Vacuolar protein sorting 39	Transporter activity	Y		
**Signalling**					
AKAP2	A kinase anchor protein 2	Cytoskeletal anchoring activity		Y	
GAK	Cyclin G associated kinase	Protein serine/threonine kinase activity		Y	
INPP5K	Skeletal muscle and kidney enriched inositol phosphatase	Lipid phosphatase activity			Y
OCRL	Phosphatidylinositol polyphosphate	Catalytic activity			Y

	5-phosphatase				
PDE4DIP	Phosphodiesterase 4D interacting protein	Transporter activity			Y
PRKRA	PKR protein activator	RNA binding			Y
PPM1G	Protein phosphatase magnesium dependent 1 gamma	Protein serine/threonine phosphatase activity		Y	Y
PPP1CA	Protein phosphatase 1, catalytic subunit, alpha isoform	Protein serine/threonine phosphatase activity		Y	
PPP2CA;PPP2CB	Protein phosphatase 2, catalytic subunit, alpha isoform	Protein serine/threonine phosphatase activity			Y
RCN1	Reticulocalbin 1	Calcium ion binding			Y
TAOK2	TAO kinase 2	Protein serine/threonine kinase activity	Y		Y
**Chaperone/Heat shock proteins**					
CCT7	Chaperonin containing T complex polypeptide 1, subunit 7	Chaperone activity			Y
DNAJA1	Heat shock 40 kDa protein 4	Heat shock protein activity	Y		
DNAJB4	DnaJ homolog subfamily B member 4	Heat shock protein activity		Y	Y
HSP90AA1	HSP90A	Chaperone activity			Y
HSP90AB2P	Heat shock protein HSP 90 beta	Chaperone activity			Y
HYOU1	Hypoxia up regulated 1	Chaperone activity			Y
STIP1	Stress induced phosphoprotein 1	Receptor signaling complex scaffold activity			Y
**RNA processing**					
AGO1;AGO2;AGO3;AGO4	Argonaute	Translation regulator activity			Y

EWSR1	Ewing sarcoma breakpoint region 1	RNA binding			Y
EPRS	Glutamyl-prolyl-tRNA synthetase	Ligase activity	Y	Y	Y
FAM120A	C9orf10 protein	RNA processing			Y
FARSB	Phenylalanyl tRNA synthetase beta chain	Ligase activity			Y
FXR2	Fragile X mental retardation syndrome related protein 2	RNA binding			Y
GRSF1	G rich RNA sequence binding factor 1	RNA binding			Y
MOV10	Moloney leukemia virus 10	RNA Helicase			Y
NCL	Nucleolin	RNA binding	Y	Y	
PABPC1;PABPC3	Polyadenylate binding protein	RNA binding	Y	Y	Y
QKI	Quaking homolog, KH domain RNA binding	RNA binding	Y		Y
SARS	Seryl-tRNA synthetase	Ligase activity	Y		Y
SRP54	Signal recognition particle 54 kDa protein	RNA binding			Y
SRPK1;SRPK2;SRPK3	SFRS protein kinase	Protein threonine/tyrosine kinase activity	Y		
ZC3H15	Immediate early response erythropoietin 4	DNA binding			Y
**Gene regulation**					
PBXIP1	Pre-B-cell leukemia transcription factor interacting protein 1	Transcription regulator activity	Y	Y	Y
RBM39	Splicing factor HCC1	Transcription regulator activity			Y
SND1	Staphylococcal nuclease domain containing protein 1	Transcription regulator activity			Y
TCEB1	Transcription elongation factor B, 1	Transcription regulator activity			Y

TFAM	Mitochondrial transcription factor 1	Transcription factor activity	Y	Y	Y
TFCP2;UBP1	Transcription factor CP2	Transcription factor activity			Y
TPP1	TIN2 interacting protein	Transcription regulator activity			Y
TRIP4	Thyroid hormone receptor interactor 4	Transcription regulator activity			Y
**Translation**					
DHX29	DEAH (Asp-Glu-Ala-His) box polypeptide 29	Helicase activity	Y		
DHX36	DEAH (Asp-Glu-Ala-His) box polypeptide 36	Helicase activity			Y
EIF3D	Eukaryotic translation initiation factor 3, subunit 7	Translation regulator activity			Y
EIF3F	Eukaryotic translation initiation factor 3, subunit 5	Translation regulator activity	Y		Y
EIF3H;EIF3S3	Eukaryotic translation initiation factor 3, subunit 3	Translation regulator activity		Y	Y
EIF4G1	Eukaryotic translation initiation factor 4 gamma, 1	Translation regulator activity	Y	Y	Y
EIF4G2	Eukaryotic translation initiation factor 4 gamma, 2	Translation regulator activity			Y
RPL10A	Ribosomal protein L10a	Structural constituent of ribosome	Y	Y	Y
RPL13A	Ribosomal protein L13a	Structural constituent of ribosome	Y	Y	
RPL18A	Ribosomal protein L18a	Structural constituent of ribosome			Y
RPL26;KRBA2	Ribosomal protein L26	Structural constituent of ribosome	Y	Y	Y
RPL27	Ribosomal protein L27	Structural constituent of ribosome	Y	Y	Y
RPL39P5;RPL39	Ribosomal protein L39	Structural constituent of ribosome	Y		Y
RPL5	Ribosomal protein L5	Structural constituent of ribosome	Y	Y	Y

RPL7	Ribosomal protein L7	Structural constituent of ribosome	Y	Y	
RPLP2	Ribosomal phosphoprotein large P2	Structural constituent of ribosome	Y	Y	Y
RPS11	Ribosomal protein S11	Structural constituent of ribosome		Y	
**Cell Metabolism**					
ACOT9	Mitochondrial acyl CoA thioesterase	Hydrolase activity	Y	Y	Y
ALDOA	Aldolase 1	Lyase activity	Y	Y	Y
CNP	2`,3` cyclic nucleotide, 3`-phosphodiesterase	Phosphoric diester hydrolase activity	Y		
DECR1	2,4-dienoyl-CoA reductase 1	Catalytic activity	Y	Y	Y
DPM1	Dolichol phosphate mannose synthase	Mannosyltransferase activity		Y	Y
GAPDH	Glyceraldehyde 3 phosphate dehydrogenase	Catalytic activity	Y		Y
GLUD1;GLUD2	Glutamate Dehydrogenase	Catalytic activity			Y
IDH2	Isocitrate dehydrogenase 2	Catalytic activity			Y
LDHA	Lactate dehydrogenase A	Catalytic activity	Y	Y	Y
P4HA2	Prolyl 4-hydroxylase, alpha polypeptide, type 2	Catalytic activity			Y
P4HB	Protein disulfide isomerase	Isomerase activity	Y		Y
PDIA6	Protein disulfide isomerase P5	Isomerase activity			Y
PGD	Phosphogluconate dehydrogenase	Catalytic activity		Y	Y
PGK1	Phosphoglycerate kinase 1	Catalytic activity			Y
PLOD3	Lysyl hydroxylase 3	Catalytic activity			Y
PRDX2	Peroxiredoxin 2	Peroxidase activity			Y
PRDX6	Peroxiredoxin 6	Peroxidase activity	Y		Y
SH3BGRL3	SH3 domain binding glutamic acid	Regulator of redox activity			Y

	rich protein like 3				
SLC25A11	Oxoglutarate carrier	Auxiliary transport protein activity	Y		Y
SYNJ1	Synaptojanin 1	Hydrolase activity	Y		
TKT	Transketolase	Transferase activity	Y	Y	Y
TXNDC5	Thioredoxin domain containing 5	Oxidoreductase activity			Y
**Other**					
ABCF2	ATP binding cassette, sub family F, member 2	Membrane transport			Y
ALB	Albumin	Transporter activity			Y
ANXA1	Annexin I	Calcium ion binding	Y	Y	Y
ARMCX1	Armadillo repeat-containing X-linked protein 1	Unknown			Y
ASCC3	ASC-1 complex subunit P200	Unknown		Y	
ATP6V0D1	ATPase H+ transporting lysosomal 38 KD V0 subunit D, isoform 1	ATPase activity			Y
ATP6V1E1	ATPase H+ transporting lysosomal subunit E	ATPase activity			Y
BSG	Basigin	Receptor activity		Y	Y
CACNA2D1	Calcium channel, voltage dependent, alpha 2/delta subunit 1	Voltage-gated ion channel activity			Y
CALR	Calreticulin	Chaperone activity			Y
CALU	Calumenin	Calcium ion binding	Y		Y
CCDC124	Coiled-coil domain containing 124	Unknown			Y
CLIC1	Chloride intracellular channel 1	Intracellular ligand-gated ion channel activity			Y
COPG1	Coatomer protein complex, subunit	Transporter activity	Y		Y

	gamma 1				
CYR61	Cysteine rich angiogenic inducer 61	Extracellular matrix structural constituent			Y
FN1	Fibronectin 1	Extracellular matrix structural constituent	Y		
GIGYF2	Grb10 interacting GYF protein 2	Unknown			Y
HBD;HBB	Hemoglobin	Transporter activity			Y
HMGB1	High mobility group box 1	DNA binding	Y		Y
HTRA1	Serine protease 11	Serine-type peptidase activity			Y
LMAN1	Lectin mannose binding 1	Chaperone activity	Y	Y	Y
LRRC59	Leucine rich repeat containing 59	Unknown	Y		Y
LRRFIP2	Leucine rich repeat (in FLII) interacting protein 2	Unknown	Y	Y	Y
LUC7L	LUC7 like	Unknown			Y
NAP1L1	Nucleosome assembly protein 1 like 1	DNA binding	Y	Y	
PRRC2C	Proline Rich Coiled-Coil 2C	Unknown			Y
PSMD11	Proteasome 26S subunit, non-ATPase, 11	Ubiquitin-specific protease activity	Y	Y	Y
PSMD4	Proteasome 26S subunit, non ATPase, 4	Ubiquitin-specific protease activity			Y
PTMA	Prothymosin alpha	Unknown	Y	Y	Y
SET	SET protein	MHC class I receptor activity	Y		Y
SSR3	Signal sequence receptor gamma	Auxiliary transport protein activity	Y		Y
SSR4	Signal sequence receptor delta	Auxiliary transport protein activity			Y
USP10	Ubiquitin specific protease 10	Ubiquitin-specific protease activity			Y

Table S3. List of proteins identified in the p130Cas WT interactome, subdivided into functional categories. Y indicates significant interaction with p130Cas at that time point
