# Supplementary material for: Vascular Endothelial Growth Factor (VEGF) Promotes Assembly of the p130Cas Interactome to Drive Endothelial Chemotactic Signaling and Angiogenesis
Source: Mol Cell Proteomics. 2016 Dec 22;16(2):168–80. doi: 10.1074/mcp.M116.064428 (PMC5294206; doi:10.1074/mcp.M116.064428)
Supplement: Supplemental Data [file 10.1074_M116.064428_mcp.M116.064428-7.pdf]

Gene Symbol	Name	Function	VEGF, min 10	30	60
**Cytoskeleton**					
AKAP2	A kinase anchor protein 2	Cytoskeletal anchoring activity	Y	Y	
CALD1	Caldesmon 1	Cytoskeletal protein binding		Y	
CEP290	Centrosomal protein, 290kDa	Structural molecule activity		Y	Y
MPRIP	Myosin phosphatase Rho interacting protein	Cytoskeletal protein binding		Y	
MYO18A	Myosin 18A	Structural molecule activity		Y	
SIPA1	Signal induced proliferation associated protein 1	GTPase activator activity	Y	Y	
SYNE2	Synaptic nuclear envelope protein 2	Structural constituent of cytoskeleton		Y	
TJP1	Tight junction protein 1	Cell adhesion molecule activity		Y	
**Actin cytoskeleton**					
ADD3	Adducin 3	Cytoskeletal protein binding	Y	Y	
ARHGEF11	Rho guanine exchange factor 11	Guanyl-nucleotide exchange factor activity	Y	Y	
CTTN	Cortactin	Cytoskeletal protein binding	Y	Y	
FLNB	Filamin B	Cytoskeletal protein binding	Y	Y	
MACF1	Macrophin 1	Cytoskeletal protein binding		Y	
PPP1R18	Protein phosphatase 1 regulatory subunit 18	Cytoskeletal protein binding	Y	Y	
RAI14	Retinoic acid induced 14	Cytoskeletal protein binding		Y	
SPTAN1	Spectrin, alpha, non-erythrocytic 1	Structural constituent of cytoskeleton	Y	Y	
SPTBN1	Spectrin beta nonerythrocytic 1	Structural constituent of cytoskeleton	Y	Y	
TRIOBP	Tara like protein	Cytoskeletal protein binding		Y	

**Cell Migration**					
CGNL1	Cingulin-like 1	Rho GTPase activity regulation	Y	Y	
CSRP2	Cysteine and glycine rich protein 2	Receptor signaling complex scaffold activity	Y	Y	
**Protein Trafficking**					
EPN1	Epsin 1	Receptor signaling complex scaffold activity	Y		
FKBP3	FK506 binding protein 3	Isomerase activity	Y	Y	
SNX18	Sorting nexin associated golgi protein 1	Transporter activity	Y	Y	
**Signalling**					
CDK11A;CDK11B	Cell division cycle 2 like 1	Protein serine/threonine kinase activity		Y	
CETN2	Caltractin	Calcium ion binding	Y		
KCTD12	Potassium channel tetramerisation domain	Ion channel activity		Y	
LEMD2	LEM domain containing 2	Nuclear membrane protein		Y	
**RNA processing**					
EPRS	Glutamyl-prolyl-tRNA synthetase	Ligase activity			Y
FARSB	Phenylalanyl tRNA synthetase beta chain	Ligase activity	Y		
PNPT1	Polyribonucleotide nucleotidyltransferase 1	Exonuclease activity	Y	Y	
SRP54	Signal recognition particle 54 kDa protein	RNA binding	Y	Y	

**Gene regulation**					
EDF1	Endothelial differentiation related factor 1	Transcription regulator activity	Y		
PA2G4	Proliferation associated protein 2G4	Transcription regulator activity	Y		
SND1	Staphylococcal nuclease domain containing protein 1	Transcription regulator activity	Y		
TCEB1	Transcription elongation factor B, 1	Transcription regulator activity	Y		
**Translation**					
ETF1	Eukaryotic translation termination factor 1	Translation regulator activity	Y	Y	
RPS17L;RPS17	Ribosomal protein S17	Structural constituent of ribosome	Y		
RPS6	Ribosomal protein S6	Structural constituent of ribosome		Y	
UPF1	Nonsense mRNA reducing factor 1	Helicase activity	Y		
**Cell Metabolism**					
SH3BGRL3	SH3 domain binding glutamic acid rich protein like 3	Regulator of redox activity	Y	Y	
**Other**					
CAPN1	Calpain, large polypeptide L1	Cysteine-type peptidase activity	Y	Y	
CTSB	Cathepsin B	Cysteine-type peptidase activity	Y	Y	Y
SAMD9L	Sterile alpha motif domain containing 9-like	Unknown		Y	
UACA	Uveal autoantigen	Unknown		Y	

Table S4. List of proteins identified in the p130Cas 15F interactome, subdivided into functional categories. Y indicates significant interaction with p130Cas at that time point
